# A study of the ground-attachment process in natural lightning with emphasis on its breakthrough phase

**DOI:** 10.1038/s41598-017-14842-7

**Published:** 2017-11-17

**Authors:** M. D. Tran, V. A. Rakov

**Affiliations:** 1 Department of Electrical and Computer Engineering, University of Florida, Gainesville, Florida 32611 USA; 2 Institute of Applied Physics, Russian Academy of Sciences, Nizhny Novgorod, 603950 Russia

## Abstract

Synchronized high-speed (124 or 210 kiloframes per second) video images and wideband electromagnetic field records of the attachment process were obtained for 4 negative strokes in natural lightning at the Lightning Observatory in Gainesville, Florida. The majority of imaged upward connecting leaders (UCLs) and upward unconnected leaders, inferred to be mostly initiated from trees, exhibited a pulsating behavior (brightening/fading cycles). UCLs, whose maximum extent ranged from 11 to 25 m, propagated in virgin air at speeds ranging from 1.8 × 10^5^ to 6.0 × 10^5^ m/s with a mean of 3.4 × 10^5^ m/s. Within about 100 m of the ground, the ratio of speeds of the downward negative leader and the corresponding positive UCL was about 3–4 for 2 events and 0.5 for 1 event. The breakthrough phase (final jump) was imaged for 2 events. The initial length of the common streamer zone (CSZ) was estimated to be about 30–40 m. For 2 events, speeds of positive and negative leaders developing toward each other inside the CSZ were found to be between 2.4 × 10^6^ and 3.7 × 10^6^ m/s. For 1 event, opposite polarity leaders were observed to accelerate inside the CSZ. The current at the end of the breakthrough phase, lasting on average 4.7 μs, was estimated to be approximately one-half of the overall current peak. Thus, about one-half of the current peak traditionally attributed to the return-stroke process is actually associated with two leaders extending toward each other to collision inside the CSZ.

## Introduction

The lightning attachment process, which can be viewed as a transition from the leader stage to the return-stroke stage, is still one of the most poorly documented lightning processes [Rakov and Uman, 2003, page 137]^1^, although considerable insights into this process have recently been made from the experiments with rocket-and-wire triggered lightning^2,3^ and long laboratory sparks^4^. It is generally assumed that the attachment process in natural lightning consists of two phases: the development of one or more upward leaders (one of which becomes the upward connecting leader (UCL)) and the so-called breakthrough phase (BTP), which is also known as the final jump. The BTP starts when the poorly-conducting streamer zones developing ahead of the hot channels of downward leader and UCL come in contact, and a common streamer zone is formed. One of the difficulties in optical imaging of the lightning attachment process is related to the fact that upward leaders and BTP are usually short (a few tens of meters or less) and faint and are followed, within a relatively short time interval, by a much brighter process, the return stroke, which commonly saturates one or more video frames.

Many researchers^5–9^ used high-speed video cameras to capture images of UCL in natural lightning terminated on towers or tall buildings, at which UCLs are generally longer. UCLs in rocket-triggered lightning were studied by Wang *et al*.^10^, Biagi *et al*.^11^, and Hill *et al*.^3^. The BTP has been imaged only for rocket-triggered lightning^3,11^ and for long laboratory sparks^4,12,13^, and in all cases, except for the one presented by Kostinskiy *et al*.^4^, the BTP was imaged in a single frame. It is worth noting that single-frame images of BTP could be influenced by the camera artifact called parasitic light sensitivity (PLS) [Tran and Rakov^14^; Hill and Mata^15^; R. Corlan and P. Martello, personal communication, 2016], which amounts to the “bleeding” of light to the BTP frame from the following frame saturated by the return stroke. Such image contamination can lead to misinterpretation of optical signatures captured immediately prior to the return-stroke onset.

The attachment process results in two current waves moving in opposite directions, one ascending toward the cloud and the other one descending toward ground^10,16,17^. The latter current wave is expected to be reflected at the ground and catch up with the upward-moving current wave to form a single, upward-moving wave^18^, which is traditionally viewed as the return stroke proper.

Jerauld *et al*.^19^ analyzed the channel-base current of a rocket-triggered lightning stroke in conjunction with electromagnetic fields recorded a few hundred meters from the channel base. They observed two loops in the lightning channel near its termination point. The current exhibited a 2.2-μs slow front (SF) from 0 to 20 kA and a 0.2-μs fast transition (FT) from 20 to 27 kA. The SF was attributed to the acceleration of downward and upward leaders as they approached each other just prior to their final connection that produced the FT in the overall current waveform. Jerauld *et al*.^19^ inferred the SF process to effectively launch a pair of kA-scale current waves propagating in opposite directions from the junction point at a speed of the order of 10^8^ m/s. Using the transmission line (TL) type models, they showed (see their Figure 11) that the SF in both close and distant field waveforms is insensitive on whether the return-stroke is assumed to start from the elevated junction point (two-wave TL model) or from ground (single-wave TL model). Nag *et al*.^20^ additionally employed a three-wave TL model (included reflection of the downward return-stroke wave from ground) and found no significant influence on the shape of SF in both close and distant field waveforms.

Nag *et al*.^20^ showed, via modeling, that the SF in electric field waveforms at far distances was primarily due to the radiation electric field component, while at near distances the contributions from electrostatic, induction, and radiation components were comparable. They also found that SF durations were similar at near and far distances, for both measured and model-predicted field waveforms. Both Jerauld *et al*.^19^ and Nag *et al*.^20^ attributed the SF in electric field to the SF in current. Nag *et al*.^20^ explicitly stated (apparently for the first time) that the mechanism of SF formation in the current is related to the BTP of the attachment process, which means that the SF process begins when the streamer zones of the downward leader and UCL come in contact and that the following acceleration of hot leader channels takes place inside the common streamer zone, until their collision which signifies the end of the SF process and the beginning of the FT process.

Howard *et al*.^2^ studied d*E*/d*t* pulses around the transition from leader to return-stroke stage in 3 natural lightning first strokes and 1 rocket-triggered-lightning stroke and identified three types of pulses (besides the regular step pulses), which they labeled the leader pulse burst (or just leader burst, LB), SF pulses, and the FT pulse. The LB was defined as a group of d*E*/d*t* pulses immediately preceding or at the onset of the SF and the SF pulses as d*E*/d*t* pulses occurring during the SF. As the name suggests, the FT pulse is the dominant d*E*/d*t* pulse during the FT part of the return-stroke waveform. In contrast with the SF and FT pulses, the LB pulses exhibited rapid movements and were prolific x-ray producers. [X-ray bursts at the time of collision of opposite polarity streamers have been observed in laboratory spark experiments^21,22^. Those observations have been followed by a number of modeling efforts^23–25^]. The FT pulse and the SF pulses were all inferred to be of the same nature and associated with multiple connections sequentially made between downward negative and upward positive leaders during the BTP. Howard *et al*.^2^ presented in their Figure 15 an optical image (video frame) showing a split triggered-lightning channel with two primary connections, one of which showing a smaller split (two subconnections), so that the total number of imaged connections was three. They related these three connections to the three major SF/FT pulses seen in their d*E*/d*t* records (see their Figure 14), which suggests that those connections were established at different times (sequentially) over the 2.1 μs duration of the BTP. Interestingly, the triggered-lightning event studied by Howard *et al*.^2^ exhibited a 20 kA abrupt increase in the channel-base current associated with a SF pulse. Another sharp current rise occurred later, at the time of the FT pulse. The overall current peak was 45 kA (unusually high for triggered-lightning strokes).

More recently, Hill *et al*.^3^ imaged the common streamer zone in rocket-triggered lightning strokes (see their Fig. 1a, 5, and 8), showed that the LB (which can be a single pulse) is associated with a fast increase from typically 10–20 A to many hundreds of amperes in the channel base current, and attributed that current increase to “the initial interactions of the downward and upward leader streamer zones”. Sources of LB pulses were located within or immediately above the connection region between the downward leader and UCL. No rapid descent of those sources was observed (in contrast with the events studied by Howard *et al*.^2^). They also related each of their SF/FT pulses with a fast, kiloampere-scale increase in the channel-base current followed by a decrease in current rate of rise. The arithmetic mean duration of the BTP in their study (measured between the abrupt current increase (onset of LB) and the FT pulse peak in the dI/dt record) was 1.77 μs, and the mean current associated with UCL just prior to LB was 16.7 A. Hill *et al*.^3^ inferred from their correlated d*E*/d*t* and d*I*/d*t* records that each SF/FT pulse was associated with a current wave propagating from the junction point (connection region) to ground. They estimated that for SF pulses the speeds of those waves were 4.3 × 10^7^ to 1.6 × 10^8^ m/s, and for FT pulses they were 1.2 × 10^8^ to 1.6 × 10^8^ m/s.

Kostinskiy *et al*.^4^ presented detailed observations of the connection between positive and negative leaders in meter-scale electric discharges generated by artificial clouds of negatively charged water droplets and discussed their implications for the attachment process in lightning. Optical images obtained with three different high-speed cameras (visible range with image enhancement (by a factor of 10^3^), visible-range regular, and infrared) and corresponding current records were used. Two frames with 100 and 50 ns exposure times separated by 2 μs, both showing the BTP, were presented for the first time. Significant branching of both leaders inside the common streamer zone was noted. Positive and negative leader speeds inside the common streamer zone were estimated for two events and found to be similar. Higher leader speeds were generally associated with higher leader currents.

Kostinskiy *et al*.^4^ suggested that a sequence of breakdowns may be involved in bridging the common streamer zone. This may happen if, after the initial connection, the impedance of the connection region and the resultant voltage drop remain sufficiently high, an additional breakdown across that region may create an additional connection, in parallel with the initial one.

In this study, we present synchronized (within better than 200 ns or so) high-speed optical and wideband electromagnetic field records and examine them with a view toward improving our understanding of the attachment process in natural lightning. Our data set consists of 4 events (see Table 1), 2 of which are presented in the main paper and 2 in the Supplementary Section S1. The dynamics of UCLs and UULs, which were imaged in multiple frames for 3 events, are studied. Optical images of the BTP (common streamer zone), observed for the first time in natural lightning, are shown for 2 events, both presented in the main paper. Upward and downward leader speeds in virgin air and in the common streamer zone are compared. Currents during the BTP are estimated using the integrated magnetic field derivative (d*B*/d*t*) waveforms and peak currents reported by the U.S. National Lightning Detection Network (NLDN). Detailed comparison of characteristics of UCLs examined in this study and those found in the literature is presented in Supplementary Section S2.

**Table 1:** Summary of one first stroke and three new-ground-termination subsequent strokes in negative lightning flashes whose attachment process was imaged at LOG in 2016. Events 1106 and 1236 are presented in the main paper and events 1238 and 1239 in Supplementary Section S1. *One of the UULs occurred before the UCL.

Event ID	Framing rate (kiloframes per second)	Exposure time (μs)	Dead time (μs)	Stroke order	Total number of strokes	Distance (km)	Spatial resolution (m)	Maximum extent of UCL (m)	UCL duration in virgin air (μs)	UCL number of frames	Number of UULs	NLDN-reported peak current (kA)
1106	124	6.36	1.69	1	6	3.4	6.4	19	—	1	0	55
1236	210	3.65	1.11	2	2	1.8	3.4	25	76	17	7	38
1238	210	3.65	1.11	3	5	2.0	3.8	11	21	4	0	7.7
1239	210	3.65	1.11	2	4	1.9	3.6	15	29	6	4*	37
Mean					4	2.3	4.3	18	42	7	3	34

## Data Presentation

### Overview

The data presented here were obtained at the Lightning Observatory in Gainesville (LOG), Florida in 2016. Description of our experimental setup is found in the section titled “Methods” below. In 2016, the Phantom camera was configured to record close (within 6 km or so) lightning channels near ground. The purpose was to study the lightning attachment process with as high time resolution as possible with our camera. A total of 4 lightning events with detectable UCLs were captured. One of these events was the first stroke and three were new-ground-termination subsequent strokes, all four transporting negative charge to ground. Pertinent information on these strokes is summarized in Table 1. According to the NLDN, all strokes terminated on ground or grounded objects within 3.5 km of the LOG. The Phantom camera was installed about 24 m above ground level (AGL) and was pointing West, where the terrain is flat and mostly covered by trees. Within the camera FoV and within 4 km radius of LOG, there was only one tall structure, which is a 76-m high communication tower at a distance of 2.7 km from the LOG. None of the four events presented here terminated on that tower. Based on the video images and NLDN data, we inferred that all the strokes examined here terminated on tree tops. The maximum heights of 10 most common (66%) tree species in the Gainesville area are between 9 and 31 m, with the average maximum height being about 18.5 m^26^. For those species, the average heights range from 6 to 27 m. In this study, we assume the lightning channel termination point to be at the bottom of the lowest luminous pixel of the imaged lightning channel trunk, although the lowest part of lightning channel could be somewhat obscured by other trees between that channel and the camera. If such tree obscuration occurs, it is expected that the actual UCL extent and duration are larger than reported here.

Of the four events presented here, three (one in the main paper and two in Supplementary Section S1) had their UCLs imaged in multiple frames, and one had its UCL seen in only one frame. Two of the three events, whose UCLs were each captured in multiple frames, also had unconnected upward leaders (UULs) imaged in multiple frames (one in the main paper and two in Supplementary Section S1). All distances and speeds estimated from optical images in this study are 2-D, which are likely to be underestimates of the actual 3-D ones by about 30%^27,28^. The camera spatial resolution was computed as Δ*l* = *r* × *d*/*f*, where *r* is the horizontal distance between the channel termination and the camera (obtained from NLDN data), *d* = 20 μm (2 × 10^−5^ m) is the pixel size, and *f* = 10.5 mm (10.5 × 10^−3^ m) is the focal length. The NLDN median location error in Florida in 2012 has been reported to be 340 m^29^ and is expected to be lower in 2016, after the most recent network upgrade completed in 2013^30^. The 4 events reported here were located by the NLDN at distances of 3.4, 1.8, 2.0, and 1.9 km from the LOG, so that the 340-m median location error translates to relative distance errors of 10%, 19%, 17%, and 18%, respectively. Since Δ*l* is proportional to *r*, the relative errors in Δ*l* are the same. [Similar results would be obtained if we used NLDN-reported semi-major axis lengths of 50% location error ellipses, which are 200, 200, 500, and 200 m for the 4 events presented here, in the same order as above.] The length of both vertical and tilted channel sections was measured between the mid-points of the upper side of the highest pixel and that of the lower side of the lowest pixel and was assigned a ±1-pixel uncertainty. In this approach, a one-pixel channel is interpreted as having a length of 0 to 2 pixels, a two-pixel channel 1 to 3 pixels, and so on. All times given in this study are relative to the initial electric field peak of the corresponding return stroke, for which we have set *t* = 0.

The 2-D channel extension rate (speed) is computed as *v* = *L*/Δ*t*, where *L* is the 2-D channel extension and Δ*t* is the corresponding time interval. The uncertainty in *L* is equal to our spatial resolution Δ*l* (as defined above); that is, to +/−1 pixel referred to the image plane. It ranges from 6% to 50% with a mean of 28% (we excluded one single-pixel speed estimate for event 1236, between −25.7 μs and −6.6 μs, with a relative error in *L* of 100%). The relative error in Δ*t*, which is computed for the worst-case timing uncertainty of 200 ns (see Supplementary Section S3), ranges from 0.3% to 17% with a mean of 2.3%. As these errors are uncorrelated, the relative error in speed measurements was computed as the square root of the sum of squares of the relative errors in time and distance measurements. The relative speed errors, which are mostly due to errors in distance measurements, range from 6% to 50% with a mean of 28% (with one event excluded, as noted above).

Next, we will present and discuss two events, 1236 and 1106, which best illustrate the salient properties of the attachment process and, in particular, its BTP. The other two events, 1239 and 1238, are presented and discussed in Supplementary Section S1 and Figs. S1, S2 and S3.

### Event 1236

Event 1236 was the second stroke of a two-stroke flash that occurred at 20:24:04 (UT) on 15 August 2016. This stroke was recorded at a distance of 1.8 km from the LOG. The first stroke of this flash was also captured by the camera but its record did not clearly show the attachment process, probably due to its larger distance (5.4 km) from the LOG. The 2-D distance between the first and second strokes was 3.9 km, so that the images of the second stroke presented here are not affected by the first stroke.

**Figure 1:**
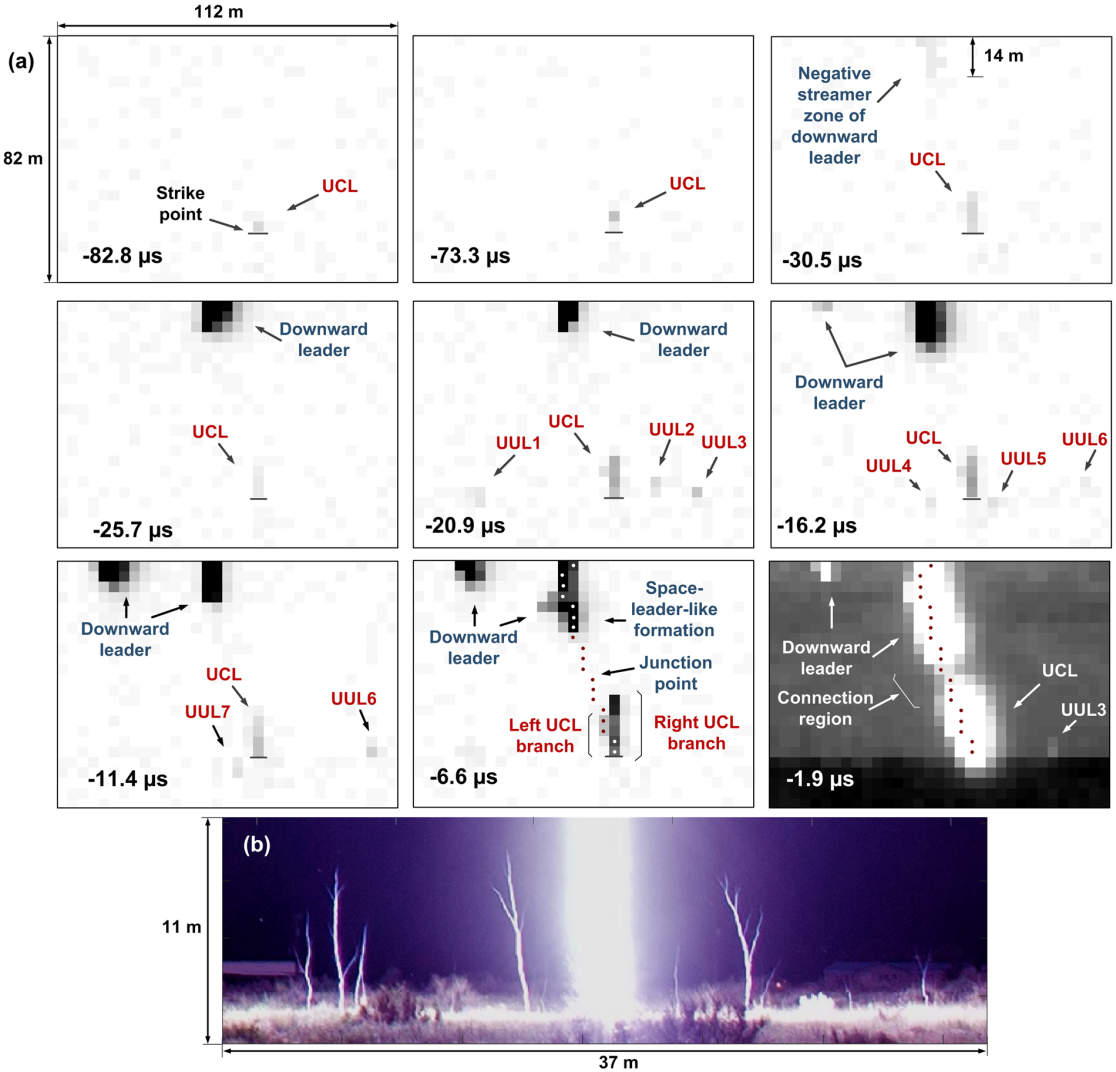
(**a**) Selected (not consecutive in the top row) frames of the attachment process of event 1236 from −82.8 to −1.9 μs showing the development of the downward leader, UCL, and 7 UULs. The end-of-exposure time is given in each frame. The interframe interval is 4.7 µs, and the pixel size is 3.4 m × 3.4 m. The UCL was first visible in frame −82.8 μs. In frames −6.6 and −1.9 μs, the non-saturated post-FT channel pixels seen at 1.4 ms are marked by red and white dots. Frame −1.9 μs immediately preceding the FT frame shows a 6.7-m non-saturated luminous region (labeled “Connection region”) between the downward leader and the UCL. All frames are processed in the same way (background-luminosity subtracted, inverted, and contrast-enhanced) for improved visualization, except for frame −1.9 μs. (**b**) Still photograph of multiple UULs near the main lightning channel observed in New Mexico. Reproduced from Cummins *et al*., Fig. 4a^31^ with permission.

Figure 1a shows selected frames between −82.8 and −1.9 μs that were cropped on the left and on the right, while preserving the full vertical FoV. The first stroke (not shown here) was completely outside of the cropped frames. The UCL was first imaged at −82.8 μs as a single luminous pixel, which faded away in the next frame (not shown in Fig. 1a). In frame −73.3 μs, the UCL brightened 2 pixels, which corresponds to a length of 6.8 m. The downward leader (DL) first entered the camera FoV in frame −35.2 μs (not shown in Fig. 1a) and slightly brightened 2 horizontally adjacent pixels at the top of the frame (61 m above the strike point). A 14-m long faintly luminous region is seen near the top of the immediately following frame at −30.5 μs, which we attribute to the negative-leader streamer zone, possibly the negative streamer corona burst produced in the step-formation process just above the camera FoV. The bright DL channel has entered the FoV at −25.7 μs, possibly causing intensification of UCL and triggering multiple UULs, as seen at −20.9 μs. [Some of the UULs could be triggered by downward stepped leader branches that were outside the camera’s FoV.] A total of 7 UULs were observed (see frames −20.9, −16.2, −11.4, and −1.9 μs). These UULs were pulsating (brightened in one frame and then faded or disappeared in the following frame). The 2-D distance between these UULs and the UCL ranged from 7 to 45 m with a mean of 21 m. Note that the origination heights of some UULs are different from that of the UCL (labeled “Strike point” in the −82.8 μs frame), which might be due to not only different heights of upward-leader-launching objects, but also due to their different distances from the camera.

The UCL in our event 1236 exhibited what we interpreted as two branches (labeled in the −6.6 μs frame of Fig. 1a). This interpretation is based on the detailed analysis of the time evolution of corresponding groups of pixels. The left UCL branch was 14 m long and became part of the main return-stroke channel. The latter is marked by red and white dots in frames −6.6 and −1.9 μs of Fig. 1a, these dots representing the return-stroke channel imaged at 1.4 ms (when there was no light blooming like in frame −1.9 μs). The right UCL branch appears to be unconnected, but could have produced a temporary connection that did not survive for 1.4 ms, during which the return stroke exhibited intense light blooming. The bottom two pixels appeared to constitute the UPL trunk, between the branching and termination points. This trunk was included in the length of each of the two branches, while computing their extension speeds, i.e. the length of each branch was measured between its tip and the assumed strike point. Using the overall duration (76.2 μs) and the corresponding length of each of the two UCL branches, the average speeds of the left and right branches were estimated to be 1.8 × 10^5^ and 2.6 × 10^5^ m/s, respectively. It is not clear why the main connection was made to the fainter and slower developing left branch.

Figure 1b is a portion of the 10-s-exposure high-resolution image of lightning obtained in New Mexico and reported by Cummins *et al*.^31^. Shown in Fig. 1b are the bottom 11 m or so of the main channel (in the center) and 12 UULs, 6 of which are branched. For these 12 UULs, the mean length and mean distance to the strike point were 4.1 m and 8.8 m, respectively. We believe that our multiple UULs, although poorly resolved, are similar to those seen in Fig. 1b.

It is interesting to compare the alternating dynamics of the left and right DL branches in frames −16.2 μs, −11.4 μs, and −6.6 μs in Fig. 1a. At −16.2 μs, the left branch (its streamer zone) just enters the FoV, while the right branch exhibits about 3-m elongation relative to the previous frame and light blooming. At −11.4 μs, the left branch shows about 7-m elongation and is blooming, while the right one does not extend and the UCL (apparently interacting with the right branch) becomes fainter. At −6.6 μs, the left branch does not extend and its blooming is reduced, while the right branch shows a step-wise extension by more than 10 m, labeled “Space-leader-like formation”, accompanied by significant intensification of UCL. We speculate that the step-wise extension facilitated both negative and positive streamer bursts (from DL and UCL tips, respectively) leading to the establishment of the common streamer zone during the dead time between frames −6.6 µs and −1.9 µs, as discussed below.

**Figure 2:**
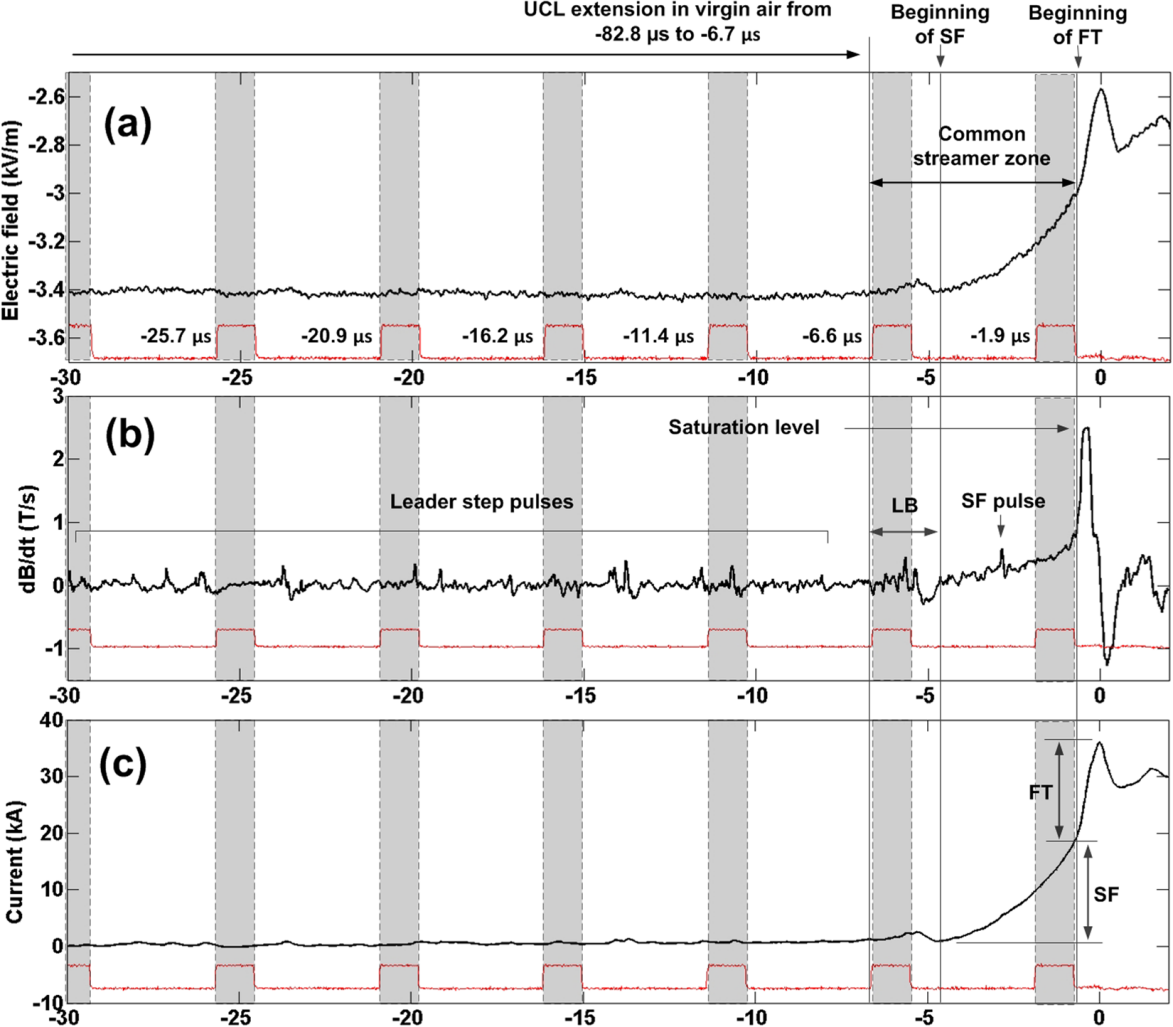
(**a**) Electric field, (**b**) d*B*/d*t*, and (**c**) inferred current of event 1236. Blank areas correspond to exposure times and shaded areas to dead times, as determined from the Strobe signal (shown in red) of the camera. The beginning of SF is marked by the beginning of the ramp in d*B*/d*t* record and is preceded by the LB. The beginning of FT is the abrupt in d*B*/d*t* rate-of-rise at the end of the SF ramp. The common streamer zone is assumed to be established at the beginning or immediately after the onset of LB and completely bridged by the hot leader channels at the end of SF (the beginning of FT). The end of exposure of frame −1.9 μs occurred after the SF pulse (marked in (**b**)), but before the end of SF. Thus, the connection region marked in frame −1.9 μs in Fig. 1 represents the common streamer zone. The d*B*/d*t* is clipped at about 2.5 T/s. The corresponding electric field waveform was used to reconstruct the current inferred from the slightly saturated d*B*/d*t* waveform.

Figure 2 shows the electric field, d*B*/d*t*, and current inferred from integrated d*B*/d*t*, with the FT pulse and other features up to 30 μs prior to it marked. The current was estimated assuming that the magnetic field and current for LB, SF, and FT are proportional to each other, as predicted by the transmission line (TL) model^32^. This approach implies that the LB, SF, and FT field signatures (the initial 5 μs or so of the return-stroke field waveform, including the LB and SF) are essentially radiation, which is supported by essentially identical shapes of the electric field and integrated dB/dt (current) fronts. The applicability of the TL model to both SF and FT was demonstrated by Jerauld *et al*.^19^ and Nag *et al*.^20^. The use of the TL model requires a value of the return-stroke speed, which is unknown. In order to avoid this difficulty, we assigned the NLDN-reported peak current to the integrated d*B*/d*t* waveform peak, so that the rest of the waveform was scaled accordingly. The median absolute error in NLDN-reported peak currents for regular subsequent strokes is 14%^29^ and it is assumed here (see work of Rakov *et al*.^33^ for a discussion of this issue) to be not much different from 14% for first and new-ground-termination subsequent strokes. Note that the d*B*/d*t* waveform for event 1236 was slightly saturated (clipped), and that the electric field waveform was used to reconstruct the corresponding B-field peak.

In this study, the beginning of SF was defined as the start of the characteristic ramp in the d*B*/d*t* record (see Fig. 2b). The LB was defined as a group of pulses or a single pulse in the d*B*/d*t* record that immediately precedes the beginnings of SF and is associated with a hump in the corresponding electric field (or inferred current) waveform. The onset of the LB was identified (somewhat subjectively) as the beginning of the first pulse that was deemed to belong to the burst. The beginning of FT was defined as the abrupt increase in the d*B*/d*t* rate-of-rise at the end of SF that culminated in the dominant d*B*/d*t* peak. The beginnings of LB, SF, and FT are marked by vertical lines in Fig. 2. Following Hill *et al*.^3^, we assume that the establishment of the common streamer zone (CSZ) occurs at the beginning of the LB (see Fig. 2b). The initial length of the CSZ is defined here as the distance between the DL and UCL tips at the onset of LB. The duration of BTP, which is characterized by the existence of common streamer zone (marked in Fig. 2a), is measured between the beginning of LB and the end of SF (or the beginning of FT) in the d*B*/d*t* waveform. In other words, the duration of BTP is the sum of durations of LB and SF. The LB was probably associated with the step-wise extension of the right branch of DL in frame −6.6 μs.

It follows from Fig. 2, where blank areas correspond to exposure times and shaded areas to dead times of the camera, that the exposure of frame −1.9 μs ended during the SF, when the current estimated from the integrated d*B*/d*t* was 9.9 kA. At the end of the SF, the current reached 19 kA, relative to the post-LB level which is not the same as the pre-LB level. The LB current hump peak was 2.4 kA. The FT duration measured from the end of SF to the initial peak in the electric field (or inferred current) waveform was 0.71 μs with the corresponding current rise being 18 kA, so that the overall current peak was 38 kA. The average speed of the DL (right branch) from −25.7 μs to −6.6 μs was 7.2 × 10^5^ m/s. For the same time interval, the speeds of the left and right branches of UCL were 1.8 × 10^5^ and 3.5 × 10^5^ m/s, respectively.

The end of exposure of frame −6.6 μs happened to be very close to the beginning of the LB. Therefore, the BTP started around the end of exposure of frame −6.6 μs, so that during the dead time and the exposure time of frame −1.9 μs the two leaders extended inside their CSZ. Between frames −6.6 and −1.9 μs, the negative and positive leaders each traveled 12 m and their frame-to-frame speeds each were 2.5 × 10^6^ m/s. [Similar speeds for oppositely charged leaders developing inside the CSZ were also reported by Kostinskiy *et al.* ^4^] The initial length of the CSZ, assumed to be equal to the distance between the tips of the DL and the left UCL branch at −6.6 μs, was 29 m. No optical evidence of CSZ is seen in frame −6.6 μs, which we interpret as resulting from either (1) its luminosity being below the camera detection level or (2) the actual CSZ onset occurring immediately after the end of exposure of frame −6.6 μs. In any event, frame −6.6 μs shows the lightning image at the very beginning of or just prior to the BTP. In frame −1.9 μs, the CSZ is seen as a relatively low-luminosity region (labeled “Connection region” in Fig. 1a) with highly-luminous (saturated) channel sections both above and below that region. The CSZ length at −1.9 μs was about 6.7 m (2 non-saturated pixels). It is worth mentioning that the connection-region luminosity in frame −1.9 μs might be influenced by the scattered light from the hot leader channels above and below it. However, the presence of CSZ at −1.9 μs is evidenced by the field and inferred current records shown in Fig. 2. We were not able to quantify the possible contribution of scattered light to the luminosity of connection region. As shown in Supplementary Section S4, the CSZ luminosity is not materially influenced by the parasitic light sensitivity (PLS) of the camera.

The rate of replacement of the relatively-high-impedance 6.7-m long CSZ seen in frame −1.9 μs by a highly-conducting channel at the end of the SF (or at the beginning of FT) was 6.3 × 10^6^ m/s. It was computed by dividing the CSZ length (6.7 m) by the time interval (1.19 μs) between the end-of-exposure of pre-FT frame (−1.9 μs) and the beginning of FT in the dB/dt record (−0.71 μs). If we assume (following Kostinskiy *et al*.^4^ and Tran and Rakov^34^) that the DL and UCL extended during the last 1.19 μs at the same speed, the speed value for each of them can be found as one-half of the replacement rate and is equal to 3.2 × 10^6^ m/s. The leaders were observed to accelerate inside the CSZ, from 7.2 × 10^5^ m/s for the negative leader and 1.8 × 10^5^ m/s for the positive leader just prior to the BTP to 2.5 × 10^6^ m/s (for each of the two leaders) from −6.6 μs to −1.9 μs and to 3.2 × 10^6^ m/s (for each of the two leaders) from −1.9 μs to −0.71 μs (the beginning of FT). These are the first speed profiles of positive and negative leaders inside the CSZ in lightning. Interestingly, the acceleration of UCL at the transition from virgin air to CSZ is considerably greater than that of DL (by a factor of 14 vs. a factor of 3.5).

### Event 1106

**Figure 3:**
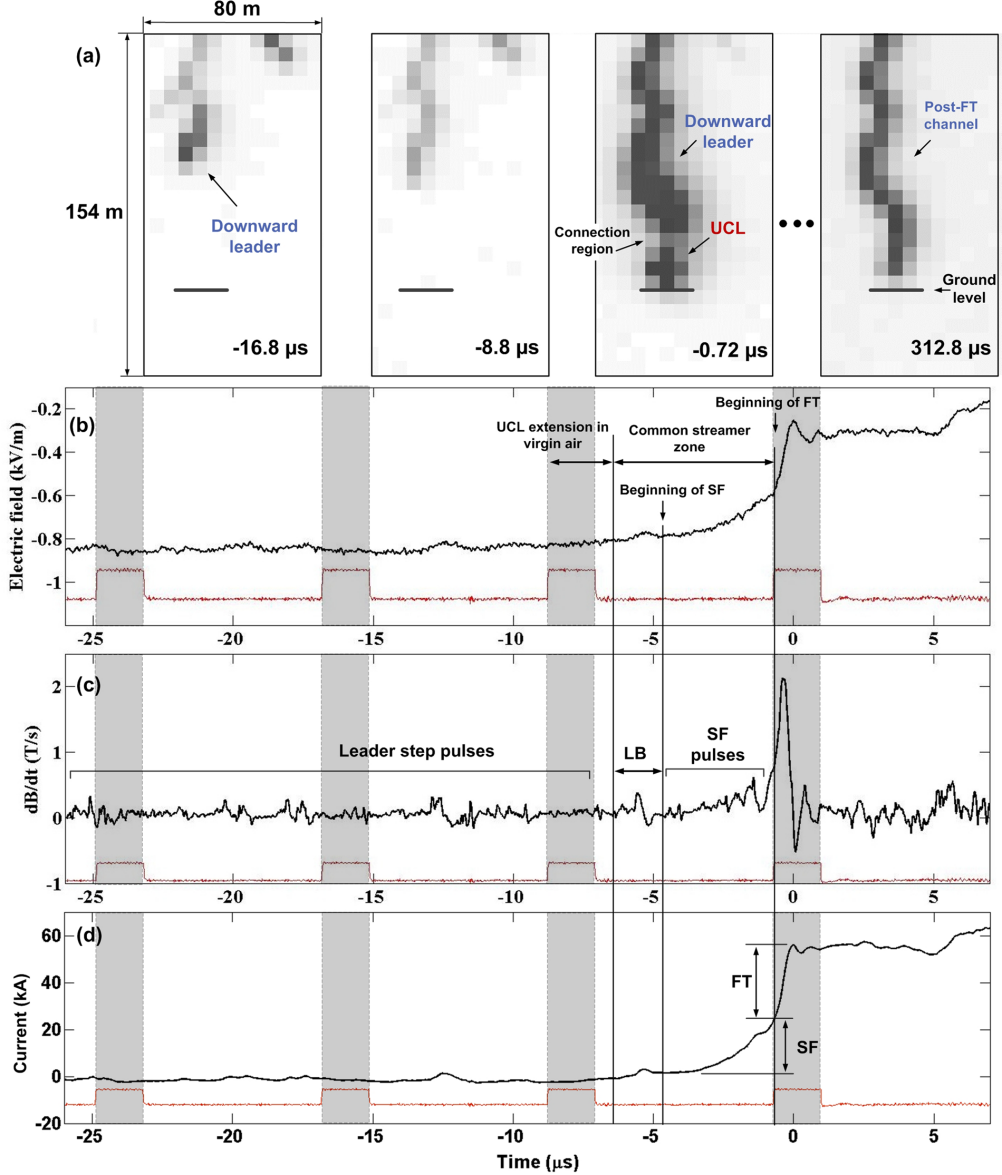
(**a**) Three consecutive frames of the attachment process of event 1106 from −16.8 to −0.72 μs showing the downward leader, UCL, and CSZ. Also shown, as a reference, is the post-FT channel at 312.8 μs. The end-of-exposure time is shown in each frame. (**b**), (**c**), and (**d**) Electric field, d*B*/d*t*, and inferred current, respectively. In (**b**–**d**), blank areas correspond to exposure times and shaded areas to dead times of the camera, as determined from the Strobe signal (shown in red). The beginnings of LB, SF, and FT are marked by vertical lines. The relatively low-luminosity pixel in frame −0.72 μs, labeled “Connection region” in (**a**), is the common streamer zone.

Figure 3a shows 3 consecutive frames, from −16.8 to −0.72 μs, as well as the post-FT channel at 312.8 μs for event 1106, which is the first stroke in a 6-stroke flash that occurred at 16:34:46 (UT) on 18 June 2016. The DL was first detected at −65.1 μs when it entered the camera FoV (not shown in Fig. 3a). Between −65.1 and −8.8 μs, the DL extended over 58 m at an average speed of 9.1 × 10^5^ m/s. From −8.8 to −0.72 μs, the DL extended over 30 m at a frame-to-frame speed of 3.7 × 10^6^ m/s. The UCL was seen only in frame −0.72 μs, where it had a length of 19 m, so that the lower bound of UCL frame-to-frame speed was 2.4 × 10^6^ m/s (we assumed that the UCL was initiated at the end of exposure of frame −8.8 µs). One can see from the field and inferred current records in Fig. 3 that the CSZ existed for about 90% of the 6.36-μs exposure time of the pre-FT (pre-initial-peak) frame or 71% of the −8.8 to −0.72 μs interframe interval. Thus, between −8.8 and −0.72 μs, the DL and UCL developed mostly inside the CSZ, which is supported by their estimated speed values of the order of 10^6^ m/s. We cannot rule out the possibility that the UCL existed in frame −8.8 μs, but was not detected due to its low luminosity and relatively large distance (3.4 km). No UULs were observed in this event.

The corresponding electric field, d*B*/d*t*, and inferred current are shown in Fig. 3b, c, and d, respectively. By “rolling back” the DL at the corresponding frame-to-frame speed (3.7 × 10^6^ m/s) and the UCL at its lower-bound speed (2.4 × 10^6^ m/s) to −6.4 μs; that is, to the onset of LB, at which the CSZ was established, the initial CSZ length was roughly estimated to be 40 m. The corresponding UCL length was about 6 m.

The peak of the current hump associated with the LB was 3.1 kA. The SF duration and current increase were 4.0 μs and 22 kA, respectively, and for the FT they were 0.71 μs and 31 kA. The current increase during the entire BTP was 24 kA, which is 44% of the overall current peak (55 kA).

The CSZ is seen in frame −0.72 μs as a faintly luminous region (6.4 m long) between the UCL and the DL, which is labeled “Connection region”. Pixels immediately above and below the connection region are saturated. The frame exposure ended around the beginning of FT, when the current was inferred to be 24 kA. Due to our timing uncertainty, the −0.72 μs frame can show either the very end of the BTP (just before the final bridging of the CSZ by hot leader channels) or the beginning of the FT process. In any event, the connection appears to be not completed at −0.72 μs, as evidenced by comparison of channel images in frames −0.72 μs and 312.8 μs (no obscuration of the channel at the position of the connection region is seen).

## Results and Discussion

In this section, we will summarize and discuss our results on the dynamics of UCLs, the extension of negative and positive leaders inside the common streamer zone, and characteristics of LB, SF, BTP, and FT. We will also compare our natural lightning results with those previously reported for rocket-triggered lightning and long laboratory sparks. Recall that our data set consists of 4 events (see Table 1), 2 of which are presented in the main paper and 2 in the Supplementary Section S1.

### Characteristics of UCLs

We obtained multi-frame optical images of UCLs and their causative downward negative leaders in lightning strikes inferred to terminate on trees of about 10–30 m in height. This is in contrast with previous high-speed video observations of the attachment process in natural lightning found in the literature, most of which, if not all, involve towers or tall buildings^5–9^ or are lacking actual images of UCLs^35^.

The maximum extents of 4 UCLs studied here ranged from 11 to 25 m with a mean of 18 m (see Table 1). The mean height of DL tip at the assumed onset of UCL (striking distance) was >53 m (median = 62 m) vs. 88 m for 3 strikes to two highly-exposed 14-story buildings, 60 m or so in height, studied by Saba *et al*.^9^. Three UCLs in the present study were observed to extend in virgin air for 21, 29, and 76 μs vs. hundreds of microseconds reported by Saba *et al*.^9^. The majority of our UCLs exhibited a pulsating behavior (brightening/fading cycles), which might have been related to downward leader branching and stepping. In two events, we also observed multiple UULs that exhibited a similar pulsating behavior. Two-dimensional speeds of UCLs and downward leaders are summarized in Table 2. UCL speeds in virgin air ranged from 1.8 × 10^5^ to 6.0 × 10^5^ m/s with a mean of 3.4 × 10^5^ m/s, which are considerably higher than those (4.3 × 10^4^ to 6.2 × 10^4^ m/s) reported by Saba *et al*.^9^ for natural lightning strikes to the 14-story buildings. In our study, for 2 events, the downward negative leaders in virgin air exhibited a factor of 3 to 4 higher speeds than the corresponding UCL did and about a factor of 2 lower speed for 1 event. Further information on UCLs studied here and on their comparison with those found in the literature is found in Supplementary Section S2. Compared to Saba *et al*.’s study^9^, our UCLs started later and propagated faster, suggesting that the electric field at the prospective strike object reached the threshold for stable leader initiation later, but the resultant UCL was more vigorous due to the proximity of the approaching DL. Further studies are needed to clarify the role of the strike object in determining the characteristics of lightning attachment process.  The UCL can be preceded by a UUL (see event 1239), which means that the initiation of upward leader does not necessarily determine the strike point.

**Table 2:** 2D speeds of downward negative leaders and UCLs, initial length of common streamer zone, and BTP current for the 4 events studied in this paper.

Event ID	Height of downward leader tip at the initiation of UCL (m)	Height of downward leader tip at the onset of common streamer zone (m)	Speed in virgin air (10^5^ m/s)			Initial length of common streamer zone (m)	Speed in common steamer zone (10^6^ m/s)			Speed immediately prior to FT^c^ (10^6^ m/s)	Current at the end of exposure of pre-FT frame (kA)
			Downward leader	UCL	Speed ratio (DL/UCL)		Downward leader	UCL	Speed ratio (DL/UCL)		
1106	59^a^	46	9.1	—	—	40	3.7	2.4	1.5	—	23
1236	>64	42	7.2	1.8	4.1	29	2.5	2.5	1.0	3.2	9.9
1238	19	—	2.8	6.0	0.5	—	—	—	—	—	2.5
1239	69	44	7.7	2.6	3.0	29	—	—	—	3.8	—
Mean	>53^b^	44	6.7	3.4	2.5	33	3.1	2.5	1.3	3.5	12
Sample size	4	3	4	3	3	3	2	2	2	2	3

^a^Assuming that the UCL was initiated at the end of exposure of frame −8.8 μs.

^b^Median = 62 m.

^c^Speeds of UCL and downward leader are assumed to be equal to each other.

For an anomalous triggered lightning flash, Wang *et al*.^17^ found that the two return stroke current waves were initiated at a height of about 23 m above the strike object (at the junction point between the downward leader and upward connecting leader), and the initiation was associated with the beginning of the SF front. They have concluded that both the SF and FT “start at the return stroke initiation height”. In our study, the heights of the downward leader tip at the time of CSZ onset could be estimated for 3 of the 4 events and ranged from 42 to 46 m, with the corresponding NLDN-reported peak currents ranging from 37 to 55 kA. For comparison, in two photoelectric studies of Wang *et al*.^17,36^ the return-stroke initiation heights for 4 first strokes were estimated to be 12 to 63 m, with the corresponding NLDN-reported peak currents being 8.9 to 76 kA.

### Characteristics of negative and positive leaders inside the common streamer zone

Frame-to-frame leader speeds inside the CSZ have been estimated for two events, 1106 and 1236, and are presented in Table 2. For event 1106, the negative leader speed is 1.5 times higher than that of the positive one. For event 1236, the speeds are the same (2.5 × 10^6^ m/s) for both polarities. For events 1236 and 1239 (the latter one is presented in Supplementary Section S1), we additionally estimated leader speeds between the end of exposure of the pre-FT frame and the end of SF (beginning of FT), based on the assumption that the positive and negative leader speeds are equal to each other. Under this assumption, the speed of each of the two leaders is equal to one-half of the final-common-streamer-zone-bridging rate, yielding similar values, 3.2 × 10^6^ and 3.8 × 10^6^ m/s for events 1236 and 1239, respectively. Interestingly, Kostinskiy *et al*.^4^, who studied electric discharges generated by artificial clouds of negatively-charged water droplets, reported that the speeds for positive and negative leaders inside the CSZ were similar, regardless of leader polarity, and ranged from 5.2 × 10^4^ to 5.5 × 10^4^ m/s. In event 1236 presented in Figs. 1a and 2, the negative leader speed increased from 7.2 × 10^5^ (in virgin air) to 2.5 × 10^6^, and then to 3.2 × 10^6^ m/s immediately prior to the FT. The positive leader (UCL) accelerated considerably faster, from 1.8 × 10^5^ (in virgin air) to 2.5 × 10^6^, and then to 3.2 × 10^6^ m/s. For both leaders, the second and third speed values were observed inside the CSZ. The ratio of DL to UCL speeds inside the CSZ was on average 1.3 (N = 2) vs. 2.5 (N = 3) in virgin air. As noted in subsection titled “Overview”, the relative errors in our speed measurements range from 6% to 50% with a mean of 28% after one single-pixel event (for which the error is ~100%) has been excluded.

Single-frame images of CSZ (that is, BTP in progress) were obtained for two events: 1106 (frame −0.72 μs in Fig. 3a) and 1236 (frame −1.9 μs in Fig. 1a). In those images, the CSZ appeared as a relatively low-luminosity region between the saturated channels of the DL and UCL.

For 3 events, the inferred currents at the end of exposure of pre-FT (pre-initial-peak) frame were 2.5, 9.9, and 23 kA, which are much higher than expected currents (usually less than a few hundreds of amperes^3,37,38^) associated with UULs and UCLs (prior to the BTP onset), but significantly lower than the corresponding overall return-stroke peak currents (7.7, 38, and 55 kA, respectively).

### Characteristics of leader burst, slow front, breakthrough phase, and fast transition

Pertinent characteristics of LB, SF, BTP, and FT are summarized in Table 3. The mean durations of LB, SF, BTP, and FT are 1.9, 2.8, 4.7, and 0.6 μs, respectively. The mean LB current hump peak is 2.1 kA, and the mean current increases during SF, BTP, and FT are 15, 16, and 18 kA, respectively. Note that the events associated with a relatively large BTP current increase tend to have a larger FT current increase. The current at the end of BTP (at the instant the CSZ is bridged by colliding hot leader channels) is approximately one-half of the overall current peak. Thus, about one-half of the current peak traditionally attributed to the return-stroke process is associated with two leaders extending toward each other to collision inside the CSZ.

**Table 3:** Characteristics of LB, SF, BTP, and FT of the 4 events studied in this paper.

Event ID	LB				SF			BTP		FT		NLDN-reported peak current^d^ (kA)
	Onset time (μs)	Duration (μs)	Current hump peak (kA)	Number of pulses	Duration (μs)	Number of superimposed pulses	Current increase^a^ (kA)	Duration^b^ (μs)	Current increase^c^ (kA)	Duration (μs)	Current increase (kA)	
1106	−6.4	1.7	3.1	2	4.0	7	22	5.7	24	0.71	31	55
1236	−6.7	2.1	2.4	5	3.9	1	19	6.0	20	0.71	18	38
1238	−3.7	2.0	0.4	2	1.3	1	3.4	3.3	3.6	0.37	4.1	7.7
1239	−4.4	1.9	2.7	4	1.9	1	16	3.8	18	0.60	19	37
Mean	−5.3	1.9	2.1	3	2.8	3	15	4.7	16	0.60	18	34

^a^SF current increase is measured relative to the current level immediately after the LB seen in the d*B*/d*t* record.

^b^BTP duration is the sum of LB and SF durations.

^c^BTP current increase is measured relative to the current level (essentially zero) immediately prior to the LB onset seen in the d*B*/d*t* record.

^d^Due to the normalization of inferred peak current to the NLDN-reported peak current, the sum of BTP and FT current increases is equal to the NDLN-reported peak current.

 The LB current hump peak is comparable to the expected step current peak of negative leaders near ground. It appears that the LB process differs from the regular leader step in that the negative corona streamer burst of the former makes contact with the positive streamer zone of grounded UCL channel, connecting the DL channel to the ground via a relatively-high-impedance common streamer zone.

Interestingly, in distant natural lightning electric field waveforms, the SF magnitude is 40% of the return-stroke field peak for both first strokes and subsequent strokes initiated by dart-stepped leaders^39^. Further, for those two types of strokes, the mean SF durations in the electric field waveforms are 4.1 and 2.1 μs, very close to, respectively, the mean BTP durations for our 4 natural lightning events (4.7 μs) and 51 rocket-triggered-lightning events studied by Hill *et al*.^3^ (1.8 μs). For comparison, for subsequent strokes initiated by dart leaders in natural lightning, the relative SF magnitude and duration are 25% and 0.9 μs, respectively^39^.

### Comparison of the attachment process in natural lightning, rocket-triggered lightning, and long laboratory sparks

Comparison of the attachment process characteristics in natural lightning [this study], in rocket-triggered lightning^3^, and in long laboratory sparks^4^ is presented in Table 4. The primary difference between rocket-triggered lightning and natural lightning, when the attachment process is concerned, is the availability of a previously-conditioned (warm-air) path to the strike point, which facilitates the occurrence of lower-peak-current strokes in rocket-triggered lightning, compared to new-ground-termination strokes in natural lightning. Laboratory sparks develop in virgin air, similar to new-ground-termination strokes in natural lightning, but occur on much smaller spatial scales. The significant differences between lightning and laboratory sparks and between first and subsequent lightning strokes in terms of the electric potential, gap length, or charge transfer are not likely to materially influence (at least qualitatively) the physical processes in the CSZ. Indeed, according to Kostinskiy *et al*.^4^, the discharge processes in the streamer zone of a leader in virgin air are determined by the electric field produced by the charges of leader tip, charges on a short segment of leader channel (including its corona sheath) just behind the tip, and charges of streamers forming the streamer zone; they only weakly depend on the large-scale external electric field produced by charges in the cloud, on leader branches (if any), etc. During the breakthrough phase, the electric field intensity inside the CSZ increases, as the two oppositely-charged hot leader channels approach each other, and the influence of the external field on the processes there becomes even less significant.

**Table 4:** Characteristics of the attachment process in natural lightning strokes [this study] vs. those in rocket-triggered lightning strokes [Hill *et al*.^3^] and in long spark discharges produced by artificial clouds of negatively charged water droplets [Kostinskiy *et al*.^4^].

Parameter	Natural lightning [this study]				Rocket-triggered lightning [Hill *et al*.^3^]				Discharges^c^ produced by artificial clouds [Kostinskiy *et al*.^4^]	
	Min	Max	AM	Sample size	Min	Max	AM	Sample size	Event 1 (Figs. 1 and 2)^d^	Event 2 (Figs. 3 and 4)^d^
Maximum UCL extent, m	11	25	18	4	3.5	11	6.9	3	~1	~1
UCL duration, μs	21	76	42	3	5.0	21	11	51	>30	~50
BTP duration, μs	3.3	6.0	4.7	4	0.8*	4.6*	1.8*	51	2.0	2.4
Initial common streamer zone length, m	29	40	33	3	~6.0	~10	~8.0	2	>0.17	>0.2
Current^a^ at the end of SF (BTP), kA^b^	3.6	24	16	4	3.6	7.8	6.2	3	5.7A	4A
Return-stroke peak current, kA^b^	7.7	55	34	4	12	15	14	3	>8A	>8A
End-of-SF current^a^ relative to peak current, %	43	52	48	4	32	53	46	3	<71	<50

^a^Corresponds to the BTP current increase in Table 3.

^b^Except for the last two columns in which currents are given in amperes.

^c^It is possible that in these discharges the upward positive leader was initiated before the onset of the downward negative leader in the cloud.

^d^References to Figs 1, 2, 3 and 4 correspond to Kostinskiy *et al*.^4^. *Additionally includes the rise time (of the order of 100 ns) of the FT pulse in dI/dt record.

In general, parameters for natural lightning in Table 4 are 2–4 times greater than their counterparts for rocket-triggered lightning. Arithmetic means of maximum UCL extent and BTP duration for rocket-triggered lightning are a factor 2 to 3 smaller than for natural lightning, while for the UCL duration and initial length of CSZ the difference is about a factor of 4. The final BTP (pre-FT) current in rocket-triggered lightning is a factor of 2 to 3 lower than in natural lightning, which is similar to the difference between the overall return-stroke peak currents.

It appears from the comparison of our results for natural lightning with those for triggered lightning^3^ and for long laboratory sparks^4^, along with other observations reviewed in the Introduction, that in each case the attachment process involves a UCL, formation of CSZ between the hot channels of downward leader and UCL, and bridging of CSZ by colliding hot leader channels. Based on that comparison, we infer that phenomenologically the attachment process of all three types of electric discharges represented in Table 4 is essentially the same.

## Summary

1. UCLs with lengths ranging from 11 to 25 m (inferred to be mostly initiated from trees) were observed using a high-speed camera operating at either 124 or 210 kiloframes per second. Both UCLs and UULs exhibited a pulsating behavior, which might be due to the stepping and branching of the descending negative leader, including alternating extension of competing branches. UCL speeds in virgin air ranged from 1.8 × 10^5^ to 6.0 × 10^5^ m/s with a mean of 3.4 × 10^5^ m/s. For 2 events, the causative downward leaders within about 100 m of the ground extended 3 to 4 times faster than the corresponding UCLs did, and in 1 event the UCL speed was twice that of the downward leader.
2. First optical images of the breakthrough phase (common streamer zone) in natural lightning were obtained for 2 events. The initial lengths of common streamer zones for 3 events were estimated to be about 30–40 m. For 1 event, a space-leader-like formation, accompanied by significant intensification of UCL, was imaged. We speculate that the step-wise extension facilitated both negative and positive streamer bursts (from DL and UCL tips, respectively) leading to the establishment of the common streamer zone. The UCL can be preceded by a UUL, which means that the initiation of upward leader does not necessarily determine the strike point.
3. First complete speed profile for colliding positive and negative leaders was estimated. Specifically, in event 1236, the negative leader speed increased from 7.2 × 10^5^ in virgin air to 2.5 × 10^6^ (by a factor of 3.5), and then to 3.2 × 10^6^ m/s just prior to the FT. The positive leader accelerated from 1.8 × 10^5^ (in virgin air) to 2.5 × 10^6^ (by a factor of 14), and then to 3.2 × 10^6^ m/s.
4. Using integrated d*B*/d*t* waveforms, NLDN-reported peak current, and a transmission-line-type model, we inferred the LB current hump peak, as well as current increases during the SF and FT. The LB current hump peak is on average about 2 kA, which is comparable to the expected step-pulse current peak in negative leaders near ground. It appears that the LB process differs from the regular leader step in that the negative corona streamer burst of the former makes contact with the positive streamer zone of grounded UCL channel, thus connecting the DL channel to the ground via a relatively-high-impedance common streamer zone. The BTP and FT current increases were on average 16 and 18 kA, respectively; that is, they contributed about equally to the overall current peak. Thus, about one-half of the current peak traditionally attributed to the return-stroke process is actually associated with two leaders extending toward each other to collision inside the CSZ.
5. Characteristics of the attachment process in natural lightning studied here were compared with their counterparts in rocket-triggered lightning and in long sparks. Arithmetic means of maximum UCL extent and BTP duration for rocket-triggered lightning were a factor 2 to 3 smaller than for natural lightning, while for the UCL duration and initial length of CSZ the difference was about a factor of 4. The final BTP (pre-FT) current in rocket-triggered lightning was a factor of 2 to 3 lower than in natural lightning, which is similar to the difference between the corresponding overall return-stroke peak currents. The BTP duration in long sparks is similar to that in rocket-triggered lightning.

## Methods

The data were obtained at the Lightning Observatory in Gainesville (LOG), Florida, which is located on the roof of a five-story building on campus of the University of Florida. The experimental setup currently includes low-gain and high-gain electric field measuring systems, electric field derivative (d*E*/d*t*) and magnetic field derivative (d*B*/d*t*) measuring systems, an x-ray detector, and 4 high-speed optical systems, a Phantom V310 camera, a Megaspeed HHC-X2 camera, a Total-Sky Imager^40^, and a LAPOS photoelectric instrument^17^. All optical data presented in this paper were obtained with the Phantom camera, whose settings were tuned to study the lightning attachment process. Additionally, data from the U.S. National Lightning Detection Network (NLDN) were used.

The useful bandwidth of the low-gain electric field measuring system is from 16 Hz to 10 MHz (decay time constant is 10 ms). For the high-gain system, it is from 360 Hz to 10 MHz, and the decay time constant is 440 *μ*s. The upper frequency response of both d*E*/d*t* and d*B*/d*t* measuring systems is 10 MHz. The electromagnetic field record lengths were 1 s with 0.4 s pretrigger. Time delays due to signal propagation along coaxial cables and fiber-optic links have been removed in presenting the waveforms. Both the optical and field records were GPS-time stamped. The uncertainty in alignment of the frame exposure time of the Phantom camera with the field records was better than 200 ns or so (see Supplementary Section S3 and Supplementary Fig. S4). The Phantom camera had 12-bit depth in its pixel value and was coupled with a 10.5-mm lens, whose relative aperture (f-number) was set to f/2.8. The camera framing rate was either 124.2 kiloframes per second (6.36 μs exposure time and 1.69 μs deadtime) or 210 kiloframes per second (3.65 μs exposure time and 1.11 μs deadtime). The corresponding frame resolutions (horizontal × vertical) were 1008 × 24 and 512 × 24 pixels, respectively. For the four recorded events, the vertical size of the field-of-view (FoV) ranged from 88 to 154 m, and the spatial resolution ranged from 3.4 to 6.4 m. The camera settings were selected to maximize the time resolution, which could be accomplished only at the expense of spatial resolution. As shown in Supplementary Section S4, our optical records are not materially affected by PLS. The camera spectral sensitivity was about 30–40% quantum efficiency (QE) in the bandwidth from 425 to 750 nm and 20–30% QE in the 750–810 nm range.

## Electronic supplementary material

Supplementary Information

## References

[1] Rakov, V. A. & Uman, M. A. *Lightning*: *Physics and Effects*, Cambridge University Press, New York (2003).

[2] Howard J (2010). RF and X-ray source locations during the lightning attachment process. Journal of Geophysical Research.

[3] Hill JD (2016). The attachment process of rocket-triggered lightning dart-stepped leaders. Journal of Geophysical Research: Atmospheres.

[4] Kostinskiy AY (2016). Observations of the connection of positive and negative leaders in meter-scale electric discharges generated by clouds of negatively charged water droplets. Journal of Geophysical Research: Atmospheres.

[5] Warner, T. A. Upward leader development from tall towers in response to downward stepped leaders. In *International Conference on Lightning Protection* (*ICLP*) Cagliari, Italy (2010).

[6] Lu W (2013). Lightning attachment process involving connection of the downward negative leader to the lateral surface of the upward connecting leader. Geophysical Research Letters.

[7] Jiang R (2015). Characteristics of lightning leader propagation and ground attachment. Journal of Geophysical Research: Atmospheres.

[8] Guimaraes, M., Arcanjo, M., Caldeira, J., Vale, M. H. M. & Visacro, S. On the features of a dart-stepped leader based on simultaneous measurements of current, E-field and high-speed video. In *24rd International Lightning Detection Conference* (San Diego, California, USA, 2016).

[9] Saba MMF (2017). Lightning attachment process to common buildings. Geophysical Research Letters.

[10] Wang D (1999). Attachment process in rocket-triggered lightning strokes. Journal of Geophysical Research: Atmospheres.

[11] Biagi, C. J. *et al*. High-speed video observations of rocket-and-wire initiated lightning. *Geophysical Research Letters* **36**, 10.1029/2009GL038525 (2009).

[12] Lebedev, V. B. *et al*. Test of the image converter cameras complex for research of discharges in long air gaps and lightning. In *13th International Conference on Atmospheric Electricity*, vol. 7, 5–8 Beijing, China (2007).

[13] Shcherbakov, Y. V. *et al*. High-speed optical studies of the long sparks in very transient stages. In *International Congress on High*-*Speed Photography andPhotonics*, edited by Hou, Xun, Zhao, Wei & Yao, Baoli Proceedings of SPIE Vol. 6279 (2007).

[14] Tran, M. D. & Rakov, V. A. Authors’ clarification to “When does the lightning attachment process actually begin?” and “Attachment process in subsequent strokes and residual channel luminosity between strokes of natural lightning”. *Newsletter on Atmospheric Electricity* **6**, http://icae.jp/newsletters/pdf/icae-vol27-1-may2016.pdf (2016).

[15] Hill, J. D. & Mata, C. T. Comments on Recent Observations of Faintly Luminous Formations (FLF) Captured Using Phantom High-Speed Cameras. In *24rd International Lightning Detection Conference* San Diego, California, USA (2016).

[16] Wang D (2013). Initiation processes of return strokes in rocket-triggered lightning. Journal of Geophysical Research: Atmospheres.

[17] Wang D (2014). Lightning attachment processes of an “anomalous” triggered lightning discharge. Journal of Geophysical Research: Atmospheres.

[18] Rakov VA (2013). The physics of lightning. Surveys in Geophysics.

[19] Jerauld, J., Uman, M. A., Rakov, V. A., Rambo, K. J. & Schnetzer, G. H. Insights into the ground attachment process of natural lightning gained from an unusual triggered-lightning stroke. *Journal of Geophysical Research*: *Atmospheres* **112**, 10.1029/2006JD007682 (2007).

[20] Nag, A. *et al*. Characteristics of the initial rising portion of near and far lightning return stroke electric field waveforms. *Atmospheric Research* **117**, 71–77, Special Issue dedicated to the 30th International Conference on Lightning Protection (ICLP) (2012).

[21] Kochkin PO, Nguyen CV, van Deursen APJ, Ebert U (2012). Experimental study of hard x-rays emitted from metre-scale positive discharges in air. Journal of Physics D: Applied Physics.

[22] Kochkin PO, van Deursen APJ, Ebert U (2015). Experimental study on hard x-rays emitted from metre-scale negative discharges in air. Journal of Physics D: Applied Physics.

[23] Kochkin P, Köhn C, Ebert U, van Deursen L (2016). Analyzing x-ray emissions from meter-scale negative discharges in ambient air. Plasma Sources Science and Technology.

[24] Ihaddadene MA, Celestin S (2015). Increase of the electric field in head-on collisions between negative and positive streamers. Geophysical Research Letters.

[25] Köhn C, Chanrion O, Neubert T (2017). Electron acceleration during streamer collisions in air. Geophysical Research Letters.

[26] Escobedo, F., Seitz, J. A. & Zipperer, W. Gainesville’s urban forest structure and composition Gainesville, Florida: School of Forest Resources and Conservation, Florida Cooperative Extension Service, Institute of Food and Agricultural Sciences, University of Florida, http://edis.ifas.ufl.edu/pdffiles/FR/FR27600.pdf (2012).

[27] Idone VP, Orville RE, Hubert P, Barret L, Eybert-Berard A (1984). Correlated observations of three triggered lightning flashes. Journal of Geophysical Research: Atmospheres.

[28] Gao Y (2014). Three-dimensional propagation characteristics of the upward connecting leaders in six negative tall-object flashes in Guangzhou. Atmospheric Research.

[29] Mallick S (2014). Performance characteristics of the NLDN for return strokes and pulses superimposed on steady currents, based on rocket-triggered lightning data acquired in Florida in 2004–2012. Journal of Geophysical Research: Atmospheres.

[30] Nag, A., Murphy, M. J., Cummins, K. L., Pifer, A. E. & Cramer, J. A. Recent Evolution of the U.S. National Lightning Detection Network. In *23rd International Lightning Detection Conference*, Tucson, Arizona, USA (2014).

[31] Cummins, K. L., Krider, E. P., Olbinski, M. & Holle, R. L. A case study of lightning attachment to flat ground showing multiple unconnected upward leaders. *Atmospheric Research*, in press, 10.1016/j.atmosres.2017.11.007 (2017).

[32] Uman MA, McLain DK (1969). Magnetic field of lightning return stroke. Journal of Geophysical Research.

[33] Rakov, V. A. *et al*. Lightning parameters for engineering applications. *CIGRE* **TB****549**, 117 (2013).

[34] Tran MD, Rakov VA (2015). When does the lightning attachment process actually begin?. Journal of Geophysical Research: Atmospheres.

[35] Stolzenburg M, Marshall TC, Karunarathne S, Karunarathna N, Orville RE (2015). Transient luminosity along negative stepped leaders in lightning. Journal of Geophysical Research: Atmospheres.

[36] Wang, D., Takagi, N., Gamerota, W. R., Uman, M. A. & Jordan, D. M. Lightning attachment processes of three natural lightning discharges. *Journal of Geophysical Research*: *Atmospheres*, 10.1002/2015JD023734 (2015).

[37] Schoene J (2008). Experimental study of lightning-induced currents in a buried loop conductor and a grounded vertical conductor. IEEE Transactions on Electromagnetic Compatibility.

[38] Visacro, S., Murta Vale, M. H., Correa, G. & Teixeira, A. Early phase of lightning currents measured in a short tower associated with direct and nearby lightning strikes. *Journal of Geophysical Research*: *Atmospheres* **115** (2010).

[39] Weidman CD, Krider EP (1978). The fine structure of lightning return stroke wave forms. Journal of Geophysical Research: Oceans.

[40] Lu, W. *et al*. Total-sky lightning event observation system and method. US20130286203, US Patent No. 8902312B2 (2014).

