# Supplementary material for: A study of the ground-attachment process in natural lightning with emphasis on its breakthrough phase
Source: Sci Rep. 2017 Nov 17;7:15761. doi: 10.1038/s41598-017-14842-7 (PMC5693988; doi:10.1038/s41598-017-14842-7)
Supplement: Supplementary file 1 — Supplementary Information [file 41598_2017_14842_MOESM1_ESM.pdf]

Supplementary Information to "A study of the ground-attachment process in natural lightning with emphasis on its breakthrough phase"

M.D. Tran[a], V.A. Rakov[a,b]

[a] *Department of Electrical and Computer Engineering, University of Florida, Gainesville, Florida, 32611, USA*
[b] *Institute of Applied Physics, Russian Academy of Sciences, Nizhny Novgorod, 603950, Russia*

S1. Data presentation for events 1239 and 1238

S1.1. Event 1239

Event 1239 was a new-ground-termination second stroke in a four-stroke flash. The first stroke was in the camera field of view (FoV), but its attachment process was not imaged, probably because of the connection region being obscured by trees. The third stroke traversed the channel of the second stroke, while the last two were out of the camera FoV (that is, at least one of them created a new, third termination on ground). The 2-D distance between the first and second strokes was 2.1 km, so that the images of the second stroke presented here are not affected by the preceding (first) stroke.

Figure S1 shows selected frames from -42.6 to 0.3 µs that were cropped on the left and on the right, while keeping the original vertical FoV. According to the NLDN, the stroke terminated 1.9 km from the LOG and its NLDN-reported peak current was 37 kA. UUL1 was the first luminous event detected within the FoV of the camera (see frame -42.6 µs in Figure S1). The main DL branch first appeared in the FoV at -37.8 µs (not shown in Figure S1) and descended between -37.8 and -4.5 µs at an average 2-D speed of 7.7 $\times 10^5$ m/s. A 7-m long, faintly luminous region is seen below the saturated DL channel tip at -14.0 µs, which probably corresponds to the streamer burst associated with the step-formation process. The UCL was first seen at -33.1 µs. A total of 4 UULs were observed in this event. The 2-D distances between these 4 UULs and the UCL ranged from 4 to 72 m with a mean of 39 m. Owing to our low spatial resolution, we were unable to determine if UUL1 and UCL had a common channel section near ground. Overall, the UCL extended over 7.3 m in 28.7 µs at an average speed of 2.6×10^5 m/s. Similar to event 1236, all the upward leaders exhibited a pulsating behavior (brightening/fading cycles).

Figure S2 shows the electric field, dB/dt, and inferred current of event 1239. The inferred current was estimated from the integrated dB/dt in the same manner as done

*Corresponding authors
Email addresses: `manhtran@ufl.edu` (M.D. Tran), `rakov@ece.ufl.edu` (V.A. Rakov)

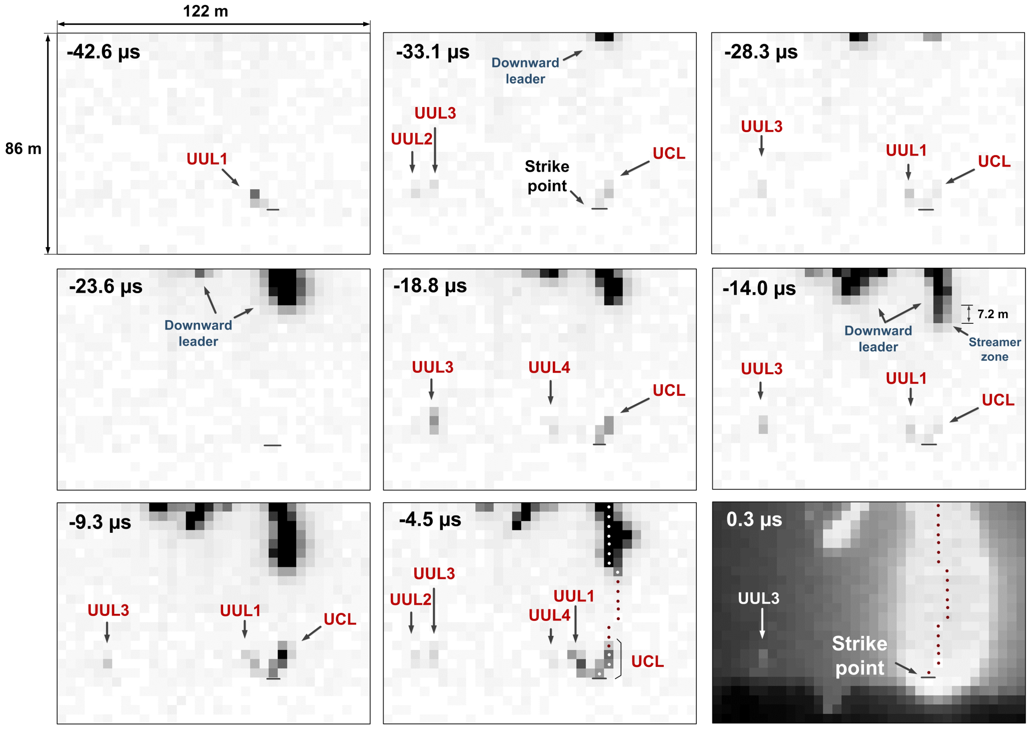

Figure S1: Selected frames (not all consecutive) of the attachment process of event 1239 from -42.6 to 0.3 µs showing the development of the downward leader, UCL, and 4 UULs. The end-of-exposure times are given in each frame. All frames are background-luminosity subtracted and enhanced for improved visualization, except for frame 0.3 µs. In frame -37.8 µs (not shown here), the downward leader branch first entered FoV, while UCL was not visible. The UCL was first imaged in frame -33.1 µs. Red and white dots in frames -4.5 and 0.3 µs correspond to the non-saturated post-FT channel, as it was seen at 1.2 ms. In frame -14.0 µs, 3 non-saturated pixels probably correspond to the 7.2 m long streamer zone below the tip of hot (saturated) leader channel.

for event 1236 (presented in the main paper). The beginnings of LB, SF, and FT are marked by vertical lines. The peak of the current hump (see Figure S2c) associated with the LB was 2.7 kA. The SF duration and current increase were, respectively, 1.9 µs and 16 kA, and for the FT those parameters were 0.6 µs and 18 kA. The overall current increase during the BTP (from the beginning of LB to the end of SF) was 19 kA.

The common streamer zone was established at -4.4 µs; that is, right after the end of exposure of frame -4.5 µs. Therefore, the 29-m long gap between the DL and UCL seen at -4.5 µs, can be viewed as the approximate initial length of the common streamer zone. If the speeds of the DL and UCL are assumed to be the same inside the common streamer zone, then the average speed of each of those two leaders during the BTP (whose duration is 3.8 µs) will be 3.8 $\times 10^6$ m/s.

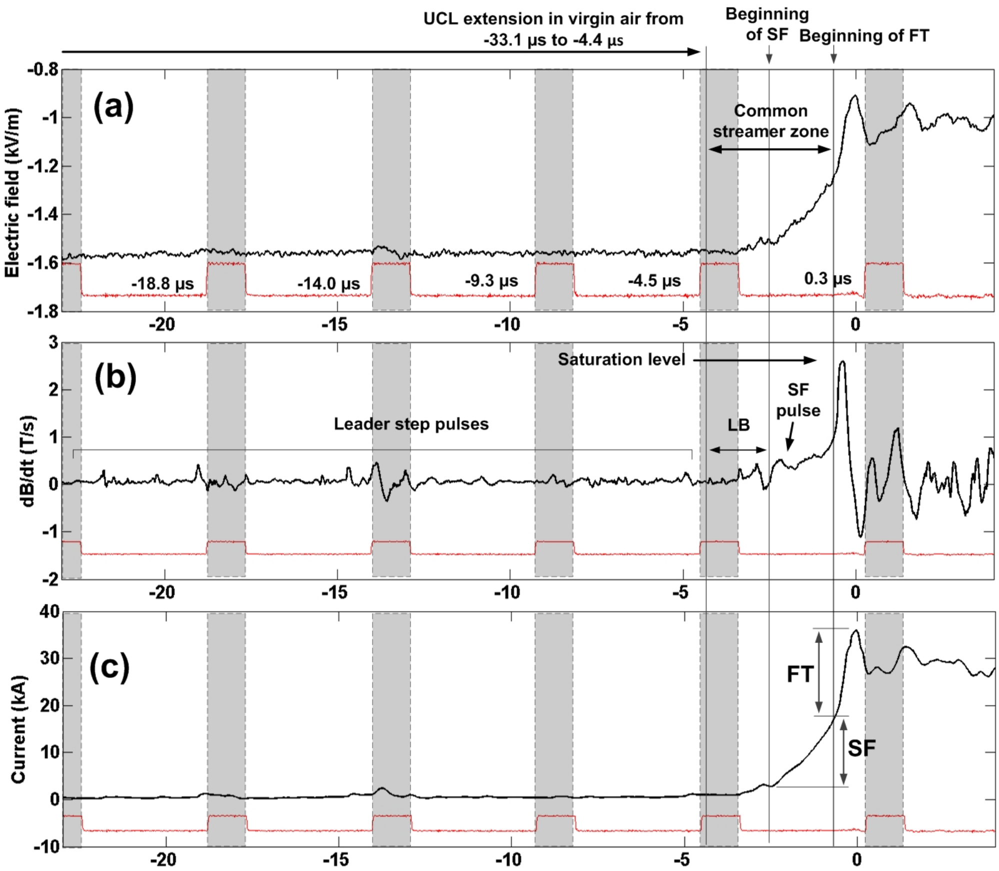

Figure S2: (a) Electric field, (b) dB/dt, and (c) inferred current of event 1239. Blank areas correspond to exposure times and shaded areas to dead times of the camera, as determined from the Strobe signal (shown in red) of the camera. Note that frame 0.3 μs contains the later part of LB, SF, and FT, so that the common streamer zone is not imaged in this case. The corresponding electric field record was used to reconstruct the current inferred from the slightly saturated dB/dt record.

S1.2. Event 1238

Event 1238 was the new-ground-termination third stroke (misclassified by the NLDN as an intracloud discharge) of a five-stroke flash. The first two strokes were out of the FoV of the camera (about 1 km from the third-stroke ground termination).

Figure S3a shows 5 consecutive frames between -19.6 and -0.6 μs and a post-FT frame at 285.1 μs. The DL was first detected at -229.2 μs (not shown in Figure S3a). Its average speed between -229.2 and -5.4 μs was 2.8 $\times 10^5$ m/s. The UCL was first seen at -24.4 μs (not shown in Figure S3a) as a single bright pixel which faded away in the following frame. At -14.9 μs, the UCL illuminated 2 pixels and appeared to extend afterwards. Its maximum extent and average speed were 11 m and 6.0 $\times 10^5$ m/s, respectively.

The corresponding electric field, $\mathrm{d}B/\mathrm{d}t$, and inferred current waveforms are presented in Figures S3b, S3c, and S3d, respectively. The beginnings of LB, SF, and FT are marked by vertical lines. The peak of the LB current hump was 0.4 kA. The SF duration and current increase were 1.3 μs and 3.4 kA, respectively, and for the FT they were 0.37 μs and 4.1 kA.

In contrast with Figure S1 and Figures 1 and 3 in the main paper the connection region is brighter than the channel above and below it, even though the exposure of frame -0.6 μs ended before the FT pulse peak. Thus, the bright connection could be associated with the pronounced SF pulse (best seen in (c)) that occurred at the very end of SF. It is also possible (due to our timing uncertainty) that the bright connection is associated with both the SF pulse and the beginning of FT, in which case the -0.6-μs frame also shows the early stage of the FT process, before its extension to the channel branching point seen in (a). The length of the highly-luminous (but not saturated) connection region at -0.6 μs was 22 m and the distance from that region to the branching point was 29 m.

S2. Comparison of characteristics of UCLs examined in this study and those found in the literature

The maximum extents of 4 UCLs examined in this paper ranged from 11 to 25 m with a mean of 18 m (see Table 1 of the paper). Three UCLs were observed to extend in virgin air for 21, 29, and 76 μs. They all exhibited a pulsating behavior (brightening/fading cycles), which is probably related to downward leader branching and stepping. This is consistent with previous observations [S1, S2, S3] that the current at grounded objects, from which UUL/UCL is initiated, exhibits pulses apparently induced by the downward negative leader. Note that Hill et al. [S3] reported UCLs detected in their channel-base current records that were not accompanied by luminosity in the corresponding optical images. Their "dark currents" were up to about 10 A.

Two-dimensional speeds of our UCLs and downward leaders are summarized in Table 2 of the main paper. UCL speeds in virgin air ranged from 1.8×10^5 to 6.0×10^5 m/s with a mean of 3.4×10^5 m/s. The speeds of their causative downward leaders in virgin air ranged from 2.8×10^5 to 9.1×10^5 m/s with a mean of 6.7 $\times10^5$ m/s. For comparison, three UCLs in previously conditioned (rocket-triggered lightning) channels reported by Hill et al. [S3] had average speeds of 2.9, 3.3, and 3.0 $\times10^5$ m/s (similar to our virgin-air UCL speeds), and corresponding final lengths of 4, 7, and 2 m (considerably shorter than maximum lengths of our UCLs, which ranged from 11 to 25 m).

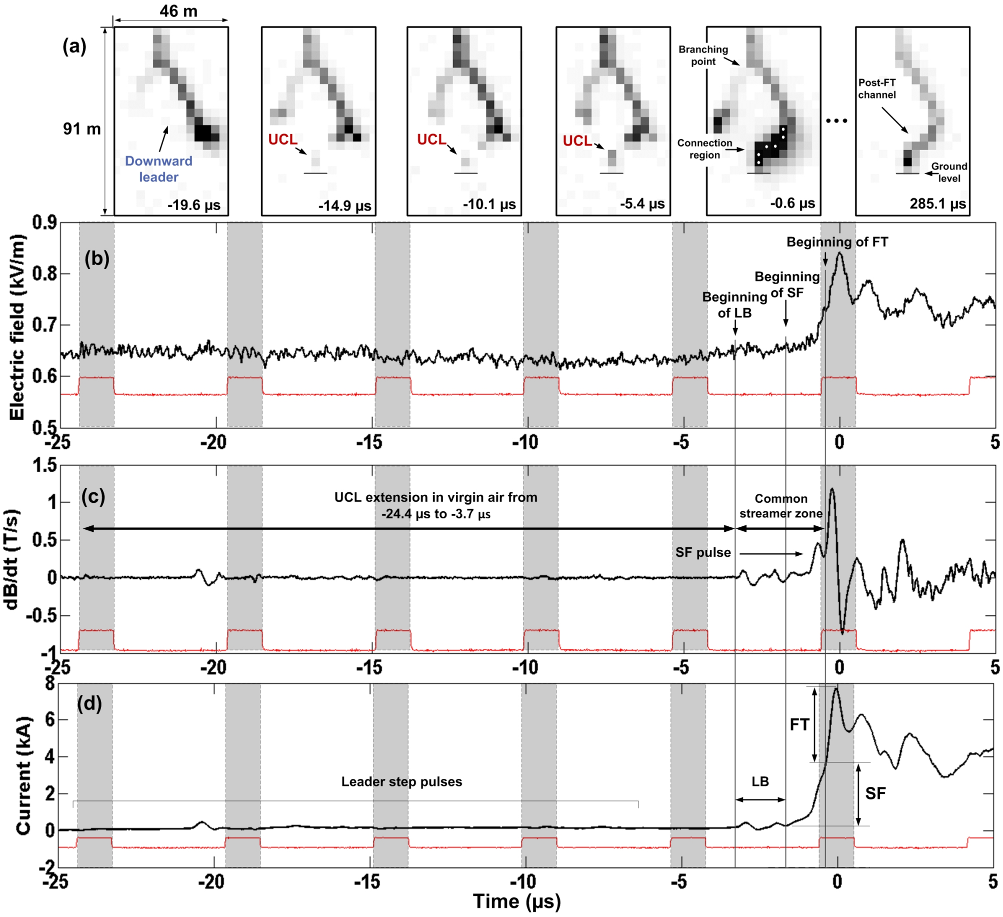

Figure S3: (a) Five consecutive frames of the attachment process of event 1238 from -19.6 to -0.6 µs showing the downward leader and UCL. Also shown, as a reference, is the post-FT channel at 285.1 µs. (b)-(d) Electric field, dB/dt, and inferred current. Blank areas correspond to exposure times and shaded areas to dead times of the camera, as determined from the Strobe signal (shown in red) of the camera.

Long (100 m or more) UCLs initiated from tall objects were observed to accelerate, although the bottom 80-90 m channel sections were too faint to allow speed estimates. Specifically, Lu et al. [S4] imaged a 400-m UCL that extended at frame-to-frame 2-D speeds ranging from 1.8×10^5 to 11.3×10^5 m/s with a mean of 4.2×10^5 m/s. Similarly, Warner et al. [S5] reported two UCLs, one of which was about 180 m long and had its 2-D speed increasing from 2.6×10^4 to 2.8×10^5 m/s, while the other one was 230 m long and had the speed increasing from 8.4×10^4 to 3.7×10^5 m/s.

For upward positive leaders (UPLs) in rocket-triggered lightning, Biagi et al. [S6] reported speeds increasing from 5.5×10^4 (over the first 11 m) to 2.1×10^5 m/s (about 185 m above the location of triggering wire top). Wang et al. [S7, Figure 5c], who examined a 320-m long UPL in natural upward lightning, reported a similar range of UPL speeds (from about 2×10^4 to 1.4×10^5 m/s). It is worth noting that UPLs can extend at speeds of the order of 10^6 m/s, when their length reaches a few kilometers and corresponding currents are of the order of kiloamperes [S8].

S3. Estimation of timing uncertainty

The camera was located 18 m from the dB/dt antenna. Therefore, the differences in time of arrival associated with different propagation paths from the lightning channel to the camera and to the dB/dt antenna could be up to 60 ns, based on the assumption that both the wideband electromagnetic field and optical signals travel at the speed of light. From the relative positions of lightning strike points, the camera, and the dB/dt antenna, we found that the signals always arrived at the camera sensor first and at the dB/dt antenna up to 60 ns later. The electric field antenna was located 4 m from the the dB/dt antenna. Therefore, the offset between the corresponding waveforms can be up to 13 ns, which is close to our sampling interval (10 ns). In our data analysis, we ignored this offset and assumed that the electric field and dB/dt waveforms are perfectly aligned. We removed one-half of the maximum (60 ns) delay in all electric field, dB/dt, and inferred current waveforms presented in this paper, so that the uncertainty due to different signal propagation paths is ±30 ns.

Another uncertainty in synchronization of our optical and field records is associated with the delay between the rising edge of the Strobe signal of the camera, corresponding to the nominal end of frame exposure, and the actual end of frame exposure. This delay was evaluated using the experimental setup shown in Figure S4a.

A signal generator feeding an LED was configured to output, with a controllable delay, a train of 10,000 short (100-ns duration and 5-ns rise-time) rectangular pulses separated by 4.76 µs when it detected a falling edge (corresponding to the beginning of frame exposure) of the camera Strobe signal. We assume here that the LED input voltage waveshape reasonably represents its output luminosity temporal profile. The fall and rise times of the Strobe signal each are 20 ns. The Strobe signal and LED input voltage were recorded by a Lecroy HDO 6000 oscilloscope. The camera was operated at a framing rate of 210 kiloframes per second (3.65 µs exposure time and 1.11 µs deadtime), which is equal to the rate of pulses in the pulse sequence at the LED input. The camera was pointed toward the LED located about 15 cm away.

By varying the delay time between the rising edge of the LED input voltage and the falling edge of the Strobe signal, we controlled the LED turn-on time relative to the beginning of frame exposure of the camera. We increased the delay time from 3.45

(just before the nominal end of exposure) to 3.95 μs (just after the nominal end of exposure) in steps of 10 ns between 3.76 and 3.83 μs and 20 ns otherwise. This delay is 3.47 μs in Figure S4. The total number of steps was 30. For each frame, we removed the background luminosity and obtained the highest pixel value in the LED image area, which we will refer to as the LED image brightness. As the delay was increased, the LED image brightness decreased and for some frames fell below the noise level when the delay was 3.76 μs (110 ns after the rising edge of the Strobe signal). The LED image brightness completely merged into the noise floor when the delay was 3.80 μs (150 ns after the rising edge of the Strobe signal). Therefore, the actual end of exposure was 110 to 150 ns after the nominal end of exposure. We can compensate for 130 ns of the delay in the end of exposure (camera's shutter transition time) with an uncertainty of ±20 ns. In order to additionally take into account the uncertainty due to the rise and fall times (20 ns) of the Strobe signal, we can compensate for 140 ns with an uncertainty of ±30 ns.

The experiment was repeated 3 times. A total of 0.9 million frames of LED images were examined and the results were essentially the same. Therefore, we conclude that after compensation for 140 ns of the bias the uncertainty in the end of exposure timing would be ±30 ns. On the other hand, the shutter transition time may be dependent on the incoming light intensity and, hence, cannot be viewed as constant. For this reason, we have decided not to introduce any compensation for the shutter transition time in our data and only discuss the effect of the 140-ns timing uncertainty (along with other uncertainties) on our results and conclusions.

Overall, we assume that our timing uncertainty was less than 60 ns (30 ns due to uncertainty in the end of exposure timing and 30 ns due to different signal propagation paths), in addition to the estimated 140-ns shutter transition time. As noted above, the latter may vary with light intensity, the dependence which we cannot check at present time. We estimate the worst-case synchronization error to be about 200 ns.

S4. Evaluation of parasitic light sensitivity effect

It is known [S9, S10, S11] that the high-speed video frame immediately preceding a very-bright-process frame can be contaminated by an attenuated image from the following, saturated frame. This camera artifact is caused by the so-called parasitic light sensitivity (PLS) of the camera, which is associated with the imperfect shielding of pixel-value storage nodes (capacitors) and their relatively long read-out time. In the case of lightning, the effect usually amounts to the "bleeding" of return-stroke light to the immediately-preceding (pre-return-stroke) frame. The intensity of resultant camera artifact in the pre-return-stroke frame is only 10^{-4} to 10^{-3} of the return-stroke intensity, but it can materially influence and even overwhelm the actual image in the pre-return-stroke frame. In the case of sparks and lightning, the artifact usually takes the form of faintly luminous formation, revealing the path of future channel extension, which can be even useful in image analysis [S9]. It is worth noting that there exist images containing faintly luminous formations in two or more consecutive frames. Examples are found in Figures 2 and 5 in Xie et al. [S12] (captured with a Photron FASTCAM SA5 camera operated at 300,000 frames per second) and in Figure 1 of Jiang et al. [S13] (captured with a Phantom V711 camera operated at 10,000 frames per second, with exposure time of 40 μs). It is not clear if those multiple-frame images are indeed influenced by PLS or show actual faintly luminous formations.

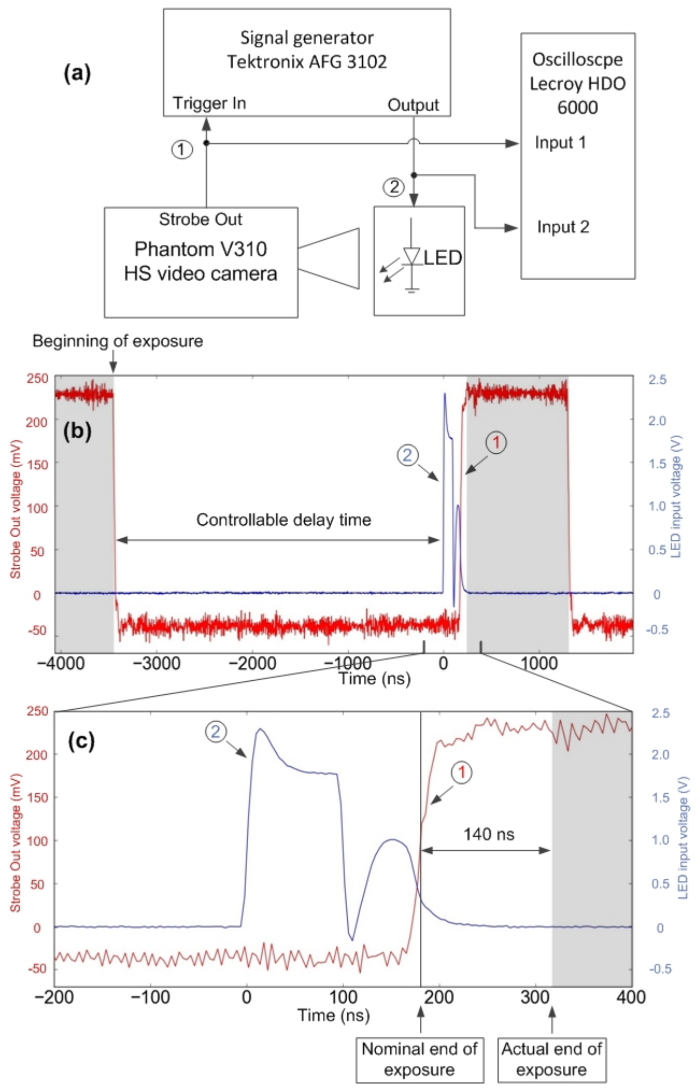

Figure S4: (a) Schematics of the experiment, in which the timing uncertainty of the end of frame exposure (tracked by the Strobe signal) was determined. (b) Strobe signal (red trace, left vertical scale) and LED input voltage (blue trace, right vertical scale). The delay between the rising edge of the LED input voltage and the falling edge of Strobe signal (corresponding to the beginning of frame exposure) was varied in the experiment and is equal to 3.470 µs in this Figure. (c) Same as (b) but shown on an expanded time scale to resolve the rising edges of the Strobe signal and LED input voltage. The oscilloscope shown in (a) recorded the two signals and was triggered by the rising edge of the LED input voltage. In (b) and (c), blank areas correspond to exposure times and shaded areas to dead times of the camera. The actual end of frame exposure was found to be delayed by 140 ns relative to the rising edge of the Strobe signal (nominal end of frame exposure). Time delays introduced by coaxial cables were removed.

In the following, we will check if the images of DLs and UCLs obtained in this study are affected by PLS and if the PLS effect, if any, can materially influence our conclusions. Specifically, we will show that 3 out of 4 UCLs are much brighter than the expected PLS-caused images, and for the fourth event the possible presence of PLS effect doesn't alter any of our conclusions. We will also show that it is unlikely that our images of common streamer zone are significantly influenced by PLS.

PLS-caused output of a pixel depends on (1) the power density of the incident light produced by the return stroke, which rapidly increases to a peak and then decreases on a longer time scale, and (2) the time during which its pixel-value storage node is exposed to the return-stroke light while waiting to be read out. We will refer to the latter as PLS exposure time or t_{PLS}. This time can be found as the time interval between the return-stroke onset and the instant the pixel-value storage node is read out. t_{PLS} can be either positive or negative. In the case of negative t_{PLS}, pixels are read out before the return-stroke onset and are not subject to PLS. For pixels that are read out after the return-stroke onset, the larger t_{PLS}, the longer the storage node exposure to return-stroke light and, hence, the stronger the PLS effect. Unfortunately, the exact value of read-out time for each pixel is unknown, which does not allow us to determin t_{PLS}. For this reason, we will use the lower bound on the read-out time and the corresponding lower bound on t_{PLS}, which we will label t_{PLS}^{min}, for each pixel of the frame. This is done based on the frequency (56 MHz) of the base clock of Phantom V310 camera [Reu and Miller [S14]; R. Corlan and P. Martello, personal communication, 2016] and the readout sequence provided by Vision Research Technical Help. The readout time of a frame in Phantom V310 camera is limited by the interframe interval, which is 4.8 and 8.05 µs for framing rates of 210 and 124.2 kiloframes per second used in the present study, respectively.

We will use PLS-caused images recorded at LOG with Phantom V310 camera in 2013 and 2014 for evaluation of PLS effect in the images acquired with the same camera in 2016 and presented in this paper. Lens settings were different in the two observation campaigns. For the same incident power density, PLS-related output is linearly proportional to the relative aperture (light-gathering area). Therefore, PLS-caused output can be converted, for a given event, from lens setting #1 to lens setting #2 using the formula given below.

$$\frac{L_1}{L_2} = \frac{A_1}{A_2} = (\frac{f_1}{f_2} \times \frac{\text{f-number}_2}{\text{f-number}_1})^2, \tag{S1}$$

where A, f, and L are the aperture area, focal length, and PLS-caused pixel output, respectively. The indices 1 and 2 correspond to lens settings #1 and #2, respectively. The PLS-caused output was computed for faintly luminous formation (FLF) events recorded in 2013-2014 with f = 20 mm and f-number = 2.8, 4, and 5.6 and then converted, using equation (S1), to the lens setting of f=10.5 mm and f-number = 2.8, that were used in 2016. Three FLF events had pixels, for which $t_{PLS}^{min} < 20$ µs. The relative PLS-caused output (in percent of the full scale) of the pixels of these three events is shown in Figure S5, in which the shaded rectangular box contains all pixels with $t_{PLS}^{min} < 10.45$ µs, the maximum readout time (8.05 µs) of the entire frame in 2016 plus the synchronization uncertainty of 2.4 µs in 2013-2014 [S15]. The maximum value of relative PLS-related output within the shaded rectangular box in Figure S5 is $\leq 5.6\%$ of the full scale.

If we were able to use t_{PLS} instead of t_{PLS}^{min}, all the data points in Figure S5 would shift to the right (and would probably all be greater than zero). Because the PLS-caused output generally tends to increase with increasing t_{PLS} (or t_{PLS}^{min}; see Figure S5), a shift of the data points to the right will likely result in a lower maximum PLS-related output.

The three events recorded in 2013 and 2014, whose PLS-related output vs. t_{PLS}^{min} scatter plot is shown in Figure S5 had NLDN-reported peak currents of 22, 25, and 99 kA, and the corresponding distances from LOG were 0.76, 3.4, and 2.0 km. These distances and peak currents are comparable to (or smaller in the case of distances and larger in the case of currents than) their counterparts for the 4 events recorded in 2016. Thus, for the same camera settings, the PLS effect associated with the 2013-2014 events was at least as significant as that for the events presented in this paper.

In the pre-FT frame of 3 events (except for event 1239) studied in this paper, the UCL channels were mostly saturated . After removing background luminosity, the relative luminosity of the UCL channels was 69, 50, and 73% for events 1106, 1236, and 1238, respectively, which are about an order of magnitude (9 - 13 times) higher than 5.6%, the upper bound of PLS-caused output with $t_{PLS} \leq 8.05$ µs. In the pre-FT image of event 1239, the relative luminosity of UCL channel was 32%, which is about 6 times higher than that expected for PLS image. Even if we stay on conservative side and accept that the PLS effect in this case is not negligible, this doesn't alter any of our conclusions, since the extents of the UCL and DL in the pre-FT frame were the same as in the preceding frame.

In summary, the UCL luminosity in the pre-FT frame of 3 out of the 4 events presented in this paper is an order of magnitude higher than the expected PLS-caused output, as estimated from previous observations. Hence, PLS did not materially influence the pre-FT images of these 3 events. The possible presence of noticeable PLS effect in the pre-FT image of the fourth event doesn't alter any of our conclusions.

Similar analysis for common streamer zone images obtained for events 1106 and 1236 indicated that their relative luminosity values are 54% and 39%, respectively; that is, a factor of 10 and 7 higher than the estimated upper bound of PLS-caused output.

Acknowledgments

We would like to thank Radu Corlan and Phil Martello of Vision Research Inc. for providing information about the Phantom V310 camera. Thanks also go to Alexander Kostinskiy who first attracted our attention to PLS effects in different high-speed video cameras.

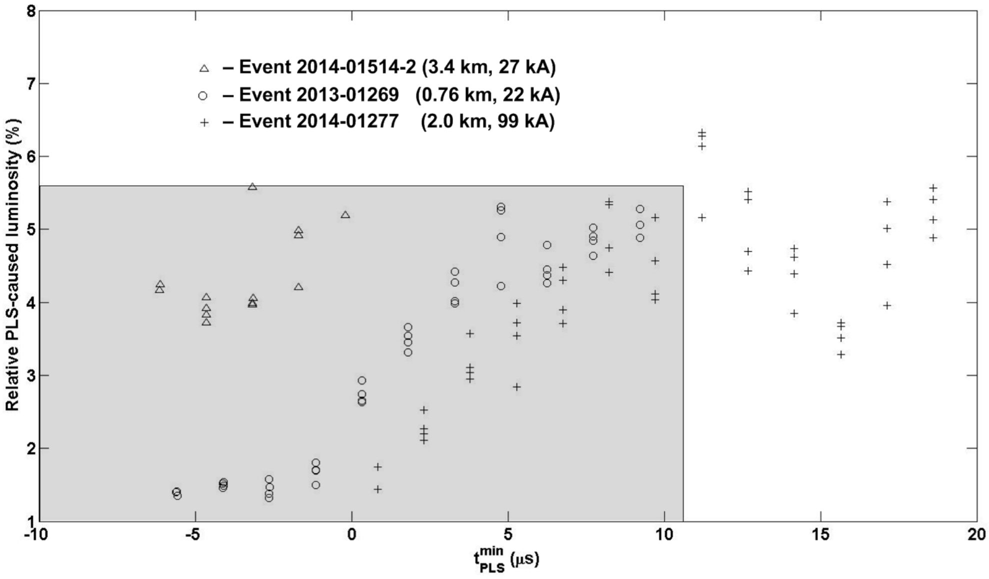

Figure S5: Relative PLS-caused luminosity (pixel output) vs. t_{PLS}^{min}, the lower bound of PLS exposure time for three faintly luminous formation events recorded in 2013 and 2014 (referred to the 2016 lens setting) that have t_{PLS}^{min} smaller than 20 μs. The shaded rectangular area contains all pixels whose t_{PLS}^{min} is less than 10.45 μs, which is chosen to be the sum of the upper bound (8.05 μs) of t_{PLS} in 2016 and the uncertainty (2.4 μs) in t_{PLS}^{min} obtained from previous observations.

Supplementary References

[S1] J. Schoene, M. A. Uman, V. A. Rakov, J. Jerauld, B. D. Hanley, K. J. Rambo, J. Howard, B. DeCarlo, Experimental study of lightning-induced currents in a buried loop conductor and a grounded vertical conductor, IEEE Transactions on Electromagnetic Compatibility 50 (1), 110–117, doi:10.1109/TEMC.2007.911927 (2008).

[S2] S. Visacro, M. H. Murta Vale, G. Correa, A. Teixeira, Early phase of lightning currents measured in a short tower associated with direct and nearby lightning strikes, Journal of Geophysical Research: Atmospheres 115 (D16), doi:10.1029/2010JD014097 (2010).

[S3] J. D. Hill, M. A. Uman, D. M. Jordan, T. Ngin, W. R. Gamerota, J. Pilkey, J. Caicedo, The attachment process of rocket-triggered lightning dart-stepped leaders, Journal of Geophysical Research: Atmospheres 121 (2) (2016) 853–871, doi:10.1002/2015JD024269 (2016).

[S4] W. Lu, L. Chen, Y. Ma, V. A. Rakov, Y. Gao, Y. Zhang, Q. Yin, Y. Zhang, Lightning attachment process involving connection of the downward negative leader to the lateral surface of the upward connecting leader, Geophysical Research Letters 40 (20) (2013) 5531–5535, doi:10.1002/2013GL058060 (2013).

[S5] T. A. Warner, Upward leader development from tall towers in response to downward stepped leaders, in: International Conference on Lightning Protection (ICLP), Cagliari, Italy (2010).

[S6] C. J. Biagi, M. A. Uman, J. D. Hill, D. M. Jordan, Observations of the initial, upward-propagating, positive leader steps in a rocket-and-wire triggered lightning discharge, Geophysical Research Letters 38 (24), doi:10.1029/2011GL049944 (2011).

[S7] Z. Wang, X. Qie, R. Jiang, C. Wang, G. Lu, Z. Sun, M. Liu, Y. Pu, High-speed video observation of stepwise propagation of a natural upward positive leader, Journal of Geophysical Research: Atmospheres 121 (24) (2016) 14,307–14,315, doi:10.1002/2016JD025605 (2016).

[S8] S. Yoshida, C. J. Biagi, V. A. Rakov, J. D. Hill, M. V. Stapleton, D. M. Jordan, M. A. Uman, T. Morimoto, T. Ushio, Z.-I. Kawasaki, M. Akita, The initial stage processes of rocket-and-wire triggered lightning as observed by VHF interferometry, Journal of Geophysical Research: Atmospheres 117 (D9), doi:10.1029/2012JD017657 (2012).

[S9] P. O. Kochkin, A. P. J. van Deursen, U. Ebert, Experimental study on hard x-rays emitted from metre-scale negative discharges in air, Journal of Physics D: Applied Physics 48 (2) (2015) 025205, doi:10.1088/0022-3727/48/2/025205 (2015).

[S10] M. D. Tran, V. A. Rakov, Authors' clarification to "When does the lightning attachment process actually begin?" and "Attachment process in subsequent strokes and residual channel luminosity between strokes of natural lightning", Newsletter on Atmospheric Electricity 6 (1) http://icae.jp/newsletters/pdf/icae-vol27-1-may2016.pdf (2016).

[S11] J. D. Hill, C. T. Mata, Comments on Recent Observations of Faintly Luminous Formations (FLF) Captured Using Phantom High-Speed Cameras, in: 24rd International Lightning Detection Conference, San Diego, California, USA (2016).

[S12] S. Xie, F. D'Alessandro, W. Chen, J. He, H. He, Attachment processes and influencing factors in competition tests under switching impulse voltages, IEEE Transactions on Plasma Science 41 (7) 1773–1780, doi:10.1109/TPS.2013.2259630 (2013).

[S13] R. Jiang, X. Qie, Z. Wang, H. Zhang, G. Lu, Z. Sun, M. Liu, X. Li, Characteristics of lightning leader propagation and ground attachment, Journal of Geophysical Research: Atmospheres 120 (23) 11,988–12,002, doi:10.1002/2015JD023519 (2015).

[S14] P. L. Reu, T. J. Miller, Synchronization Errors in High-Speed Digital Image Correlation, in: Proceedings of the SEM Annual Conference, Albuquerque, New Mexico, USA (2009).

[S15] M. D. Tran, V. A. Rakov, When does the lightning attachment process actually begin?, Journal of Geophysical Research: Atmospheres 120 (14) 6922–6936, doi:10.1002/2015JD023155 (2015).
